# Controlled Growth of Highly Defected Zirconium–Metal–Organic Frameworks via a Reaction–Diffusion System for Water Remediation

**DOI:** 10.1021/acsami.3c16327

**Published:** 2024-01-17

**Authors:** Patrick Damacet, Karen Hannouche, Abdelaziz Gouda, Mohamad Hmadeh

**Affiliations:** † Department of Chemistry, Faculty of Arts and Sciences, American University of Beirut, Beirut 1107 2020, Lebanon; ‡ Department of Chemistry, Burke Laboratory, Dartmouth College, Hanover, New Hampshire 03755, United States; § Department of Chemistry, University of Toronto, 80 St. George Street, M5S 3H6 Toronto, Canada

**Keywords:** Crystal growth, metal−organic frameworks, reaction diffusion process, defects control, Zr-MOFs, methylene blue, adsorption

## Abstract

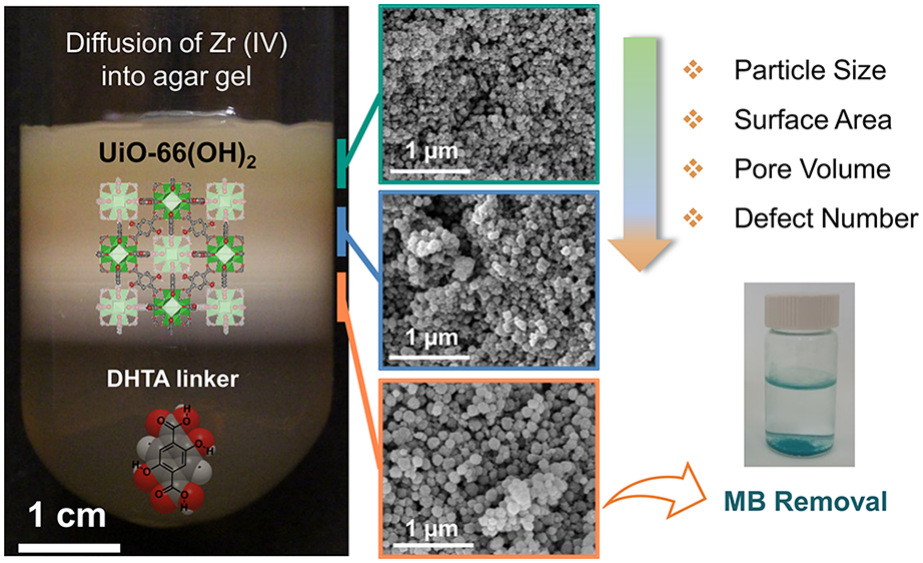

The relentless growth of metal–organic framework (MOF) chemistry is paralleled by the persistent urge to control the MOFs physical and chemical properties. While this control is mostly achieved by solvothermal syntheses, room temperature procedures stand out as more convenient and sustainable pathways for the production of MOF materials. Herein, a novel approach to control the crystal size and defect numbers of a dihydroxy-functionalized zirconium-based metal–organic framework (UiO-66(OH)_2_) at room temperature is reported. Through a reaction–diffusion method in a 1D system, zirconium salt was diffused into an agar gel matrix containing the organic linker to form nanocrystals of UiO-66(OH)_2_ with tailored structural features that include crystal size distribution, surface area, and defect number. By variation of the synthesis parameters of the system, hierarchical MOF nanocrystals with an average size ranging from 30 nm up to 270 nm and surface areas between 201 and 500 m^2^ g^–1^ were obtained in a one-pot synthetic route. To stress the importance of crystal size, morphology, and structural defects on the adsorption properties of UiO-66(OH)_2_, the adsorption capacity of the MOF toward methylene blue dye was tested with the largest and most defected crystals achieving the best performance of 202 mg/g. The distinctive structural characteristics including the hierarchical micromesoporous frameworks, the nanosized particles, and the highly defective crystals obtained by our synthesis procedure are deemed challenging through the conventional synthesis methods. This work paves the way for engineering MOF crystals with tunable physical and chemical properties, using a green synthesis procedure, for their advantageous use in many desirable applications.

## 1 Introduction

Metal–organic frameworks (MOFs) have received immense attention as a promising class of crystalline porous materials in reticular chemistry.^1−3^ MOFs are generally composed of polytopic organic linkers connected by metal nodes to form extended periodic network structures.^4^ Their unique structural properties, including high surface area, chemical and thermal stability, tunability, and crystallinity have rendered them attractive candidates in an array of applications including gas separation,^5,6^ ion filtration,^7^ catalysis,^8−10^ chemical sensing,^11,12^ and antibacterial applications.^13,14^

As many MOF materials are currently being explored as potential candidates for commercial applications, careful regard is being directed toward the generation of robust multifunctional MOFs suitable for practical and real-world applications.^15−18^ UiO-66 (University of Oslo) MOF is among the foremost classes of MOFs with outstanding chemical and mechanical properties well retained under harsh environments.^19^ First reported in 2008, UiO-66 is composed of hexa-nuclear Zr(IV) clusters cross-linked by 12 terephthalate-based linkers resulting in a 3D network with permanent porosity and large surface area.^20^ Credited to its high connectivity and strong Zr–O chemical bonds within the cluster, UiO-66 crystals can withstand structural deformations through defect engineering strategies, resulting in a versatile framework with both Brønsted and Lewis acid sites for adsorption and catalysis applications.^21^

The size of MOF crystals represents an element of control, beyond chemical compositions, for exploiting the mechanochemical properties of the framework at the mesoscale.^22^ For Zr-MOFs, it is possible to control the crystal size via a modulation approach encompassing the addition of a monocarboxylic acid, which actively competes with the ligand on coordinating to the metal nodes, slowing down the rates of nucleation and growth of the MOF.^23^ Despite being the most widely used method in controlling particle size distribution in UiO-66 systems, this method requires solvothermal synthesis conditions in addition to high activation temperatures which exceed 120 °C in most cases, hence resulting in high production costs attributed to the large energy and time consumption.^24,25^ Other attempts to control the crystal size in the bulk phase have evolved around employing a solvent evaporation method using two types of modulators.^26^ This method nonetheless resulted in the formation of agglomerates of Zr-MOF particles with sizes ranging from a few nanometers to a couple of micrometers in the same reaction medium.

An alternative way to tune the size of MOF crystals involves a direct control over their mechanisms of nucleation and growth.^27^ These processes are hard to monitor in solvothermal methods especially with UiO-66 where the reaction between the terephthalic acid and the Zr(IV) metal nodes is very fast making it challenging to lengthen the nucleation process.^28^ Recently, our research group demonstrated that MOF materials with controlled crystal size and morphology can be generated in a hydrogel medium via self-assembly while relying on the diffusion process of metal cations and linkers.^29,30^ This reaction–diffusion approach comprises the addition of an outer electrolyte solution, constituted of one of the MOF’s starting materials, on top of a gel matrix containing the other building block. The resulting diffusion flux, formed at the liquid–gel interface, is proportional to the concentration gradient, which in turns affects the morphological and textural properties of the crystals along the tube. In fact, the intertwining effect of nucleation and crystal growth rates dictates the size of the extracted crystals with smaller ones obtained near the interface, where nucleation dominates, while larger particles gradually form down the tube because of crystal growth domination.^29^

Herein, we report the synthesis of a dihydroxy-functionalized UiO-66 framework, denoted by UiO-66(OH)_2_-Z (Z = zone number), via a reaction–diffusion process at room temperature. This method offers three distinctive advantages over the standard solvothermal technique, which is commonly utilized for the preparation of UiO-66 materials. First, it allows for precise control over the size, defect number, and surface area of the MOF crystals by adjusting the reaction’s chemical parameters. Second, it is carried out at room temperature with no thermal treatments and a limited usage of hazardous solvents, hence resulting in low energy costs and minimal environmental impact and carbon footprint. Third, it offers a means to examine the effect of various experimental parameters on the MOF particle growth. As proof-of-concept application, we also demonstrate the role of particle size and defects present in this newly synthesized UiO-66(OH)_2_ on the adsorptive removal of methylene blue from solution. This study provides a green, controlled, and refined synthesis approach of one of the most remarkably stable MOFs, with well-established synthesis trends allowing the facile tuning of crystals properties. These properties are deemed to be essential for the enhancement of MOFs adsorption capacities, thus unraveling the potential of this engineering approach in water treatment and other industrial applications.

## 2 Methodology

In this study, dihydroxy-functionalized UiO-66, denoted as UiO-66(OH)_2_ was taken as a model system for the broader class of Zr-MOFs for a variety of reasons. First, it is one of the most studied and reported Zr-MOF systems in literature.^31−33^ Second, it is widely investigated in water remediation and gas adsorption/separation applications in addition to being excessively incorporated in mixed matrix membranes.^31−34^

### Synthesis Method

**Figure 1:**
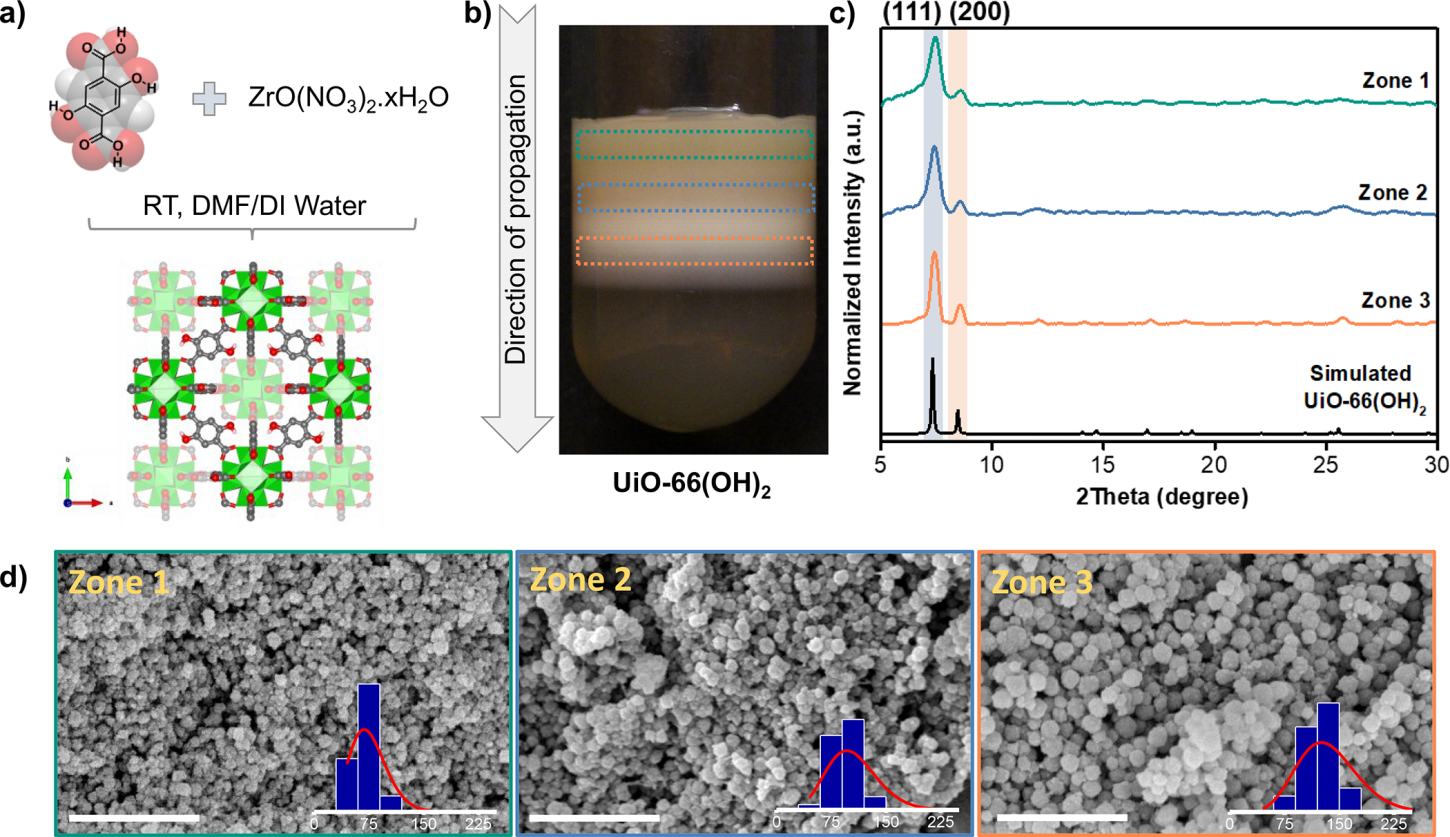
(a) Synthetic scheme and crystal structure of UiO-66(OH)_2_ indicating the coordination of the zirconium clusters to the 2,5-dihydroxyterephthalic acid ligands. Zirconium clusters are represented in green, carbon atoms in gray, and oxygen atoms in red. Hydrogen atoms have been omitted for clarity. (b) A photograph profile of UiO-66(OH)_2_ prepared in a test tube showing the diffusion of the outer electrolyte (zirconium nitrate) into a gel matrix made up of acetic acid, terephthalic acid derivative, and agar gel. Three zones were extracted from the reaction tube. (c) PXRD patterns of the MOF particles extracted from all zones along with their characteristic crystallographic planes. (d) SEM images of the MOF particles extracted from the three reaction zones. Inset histograms represent the MOF particle size distribution. Note that the SEM scale bar is 1 μm.

UiO-66(OH)_2_ MOF was generated at room temperature using a reaction–diffusion process by diffusing an outer electrolyte solution, made up of zirconium(IV) nitrate hydrate dissolved in a mixture of water and dimethylformamide, on top of an agar gel matrix containing a 2,5-dihydroxyterephthalic acid linker and acetic acid modulator in a test tube (Figure 1a and b and Figure S1). While several types of gelling agents have been previously reported in reaction–diffusion systems, gelatin-like substances were found to be the most suitable to grow crystals of inorganic compounds due to their high porosity, mechanical strength, and solubility in water.^35,36^ After the test tube was left undisturbed for 7 days to allow the outer electrolyte to propagate into the gel, the reaction zone where the MOF crystals formed was extracted from the tube via a sliding operation with a stainless steel spatula and divided into three equidistant zones of 1 cm each. MOF crystals extracted from all zones were washed with hot deionized water to dissolve the gel, followed by washing with acetone, before finally being activated in a vacuum oven overnight. More information on the synthesis procedure can be found in Section 1 of the Supporting Information.

### Methylene Blue (MB) Adsorption Experiments

Proof-of-concept adsorption experiments were performed to validate the effect of controlling the particle size of UiO-66(OH)_2_ on the adsorption properties of the framework. Methylene blue (MB) was utilized as a model organic pollutant due to its low biodegradability and toxicity in water. All adsorption experiments were performed at room temperature in 20 mL scintillation vials using a Daihan orbital shaker (ShkO-300). In brief, 5 mg of the respective MOF material was added to 10 mL of MB solutions of different initial concentrations for 5 h. The MOF powder was then filtered from the solutions using 0.45 μm syringe filters, and the supernatant was analyzed via UV–vis spectroscopy to determine the remaining concentration of MB.

The equilibrium adsorption capacity *Q*_e_ (mg_MB_/g_MOF_) at a given initial concentration of each aliquot for a given MOF sample was determined by using eq 1:
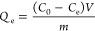
1where *C*_0_ is the initial concentration of MB (in ppm), *C*_e_ is the concentration of MB remaining in the solution after adsorption (in ppm), *V* is the volume of the solution (in mL), and *m* is the mass of the MOF sample used (in mg).

Furthermore, a kinetic study was performed in which the amount of MB left in solution after ion adsorption was determined at different time intervals to assess the adsorption performance and uptake capacity of MB. Calculations were determined according to eq 2:
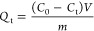
2where *Q*_t_ is the adsorption uptake at a particular time (in mg_MB_/g_MOF_), and *C*_t_ is the concentration of MB remaining in the solution at a given time (in ppm).

The effect of pH on the adsorption uptake of the MOF samples at a fixed concentration of 100 ppm was also examined. The pH of each solution was adjusted by adding hydrochloric acid (HCl) or sodium hydroxide (NaOH) to decrease or increase the pH, respectively. Then, the adsorption experiments were carried out, and the equilibrium adsorption capacity *Q*_e_ was determined by calculations according to eq 1.

## 3 Results and Discussion

### Characteristics and Properties of MOF Crystals

For the initial reaction–diffusion system, the concentrations of the inner and outer reactants used were 20 and 200 mM, respectively, whereas the agar gel percentage was chosen to be 1% w/w. These conditions were chosen based on several optimization reactions initially carried out. Following the formation of the MOF crystals in the gel, three zones were extracted, washed vigorously, and analyzed via powder X-ray diffraction (PXRD), scanning electron microscopy with energy dispersive X-ray spectroscopy (SEM/EDX), transmission electron microscopy (TEM), thermogravimetric analysis (TGA), and Brunauer–Emmett–Teller (BET) surface area analyzer.

PXRD analysis allowed us to assess the phase purity and crystallinity of the framework materials extracted from all zones. As illustrated in Figure 1c, the diffraction patterns of the synthesized materials were found to be well resolved and in good agreement with the simulated patterns for UiO-66(OH)_2_. Interestingly, the diffraction pattern for the (111) crystallographic plane was found to be broad for the MOF particles formed in zone 1 (near the liquid–gel junction) compared to zones 2 and 3 (down the reaction tube), which became narrower, more intense, and highly resolved. This is consistent with Scherrer’s equation which indicates that small crystallites produce broad peaks whereas large ones result in narrower peaks (eq S1).^37^ The calculated average particle sizes for each zone are shown in Table S1 and indicated a near 2-fold increase in the average particle size distribution from 74 nm in zone 1 to 134 cm in zone 3. This increase in the particle size along the tubular reactor is a characteristic of the reaction diffusion process, whereby the supersaturation gradient is the main contributor to the spatial distribution of the crystal sizes. SEM and TEM were then employed to evaluate the average size distribution and assess the morphology of the obtained crystals. For all zones, UiO-66(OH)_2_ particles assembled as nanosized irregular grains to form hierarchical structures as seen in Figure S2 as opposed to those synthesized via solvothermal means which are known to display a polyhedral morphology.^33^ Furthermore, the growth and evolution of the MOF crystals in the reaction tube were examined. Going down the reaction tube through different zones, zone 1 seemed to enclose the smallest pseudospherical crystals where nucleation is dominating and the supersaturation wave is at its highest, whereas zone 3, located at the bottom of the reaction zone, accommodated the largest crystals due to the low intensity of the concentration gradient at that point, allowing the growth of MOF crystals. Moreover, quantitative EDX analysis for the largest obtained particles matched previous reports of UiO-66(OH)_2_ (Figure S3).^38^ The average particle size in each zone was further analyzed and compared with the estimated values extracted from the Scherrer equation. The average particle size determined from SEM analysis increased by two folds from 58 nm in zone 1 to 119 nm in zone 3 (Figure 1d), confirming the advantages that the reaction–diffusion system offers in synthesizing UiO-66(OH)_2_ with different particle sizes, uniformly distributed in each zone.

**Figure 2:**
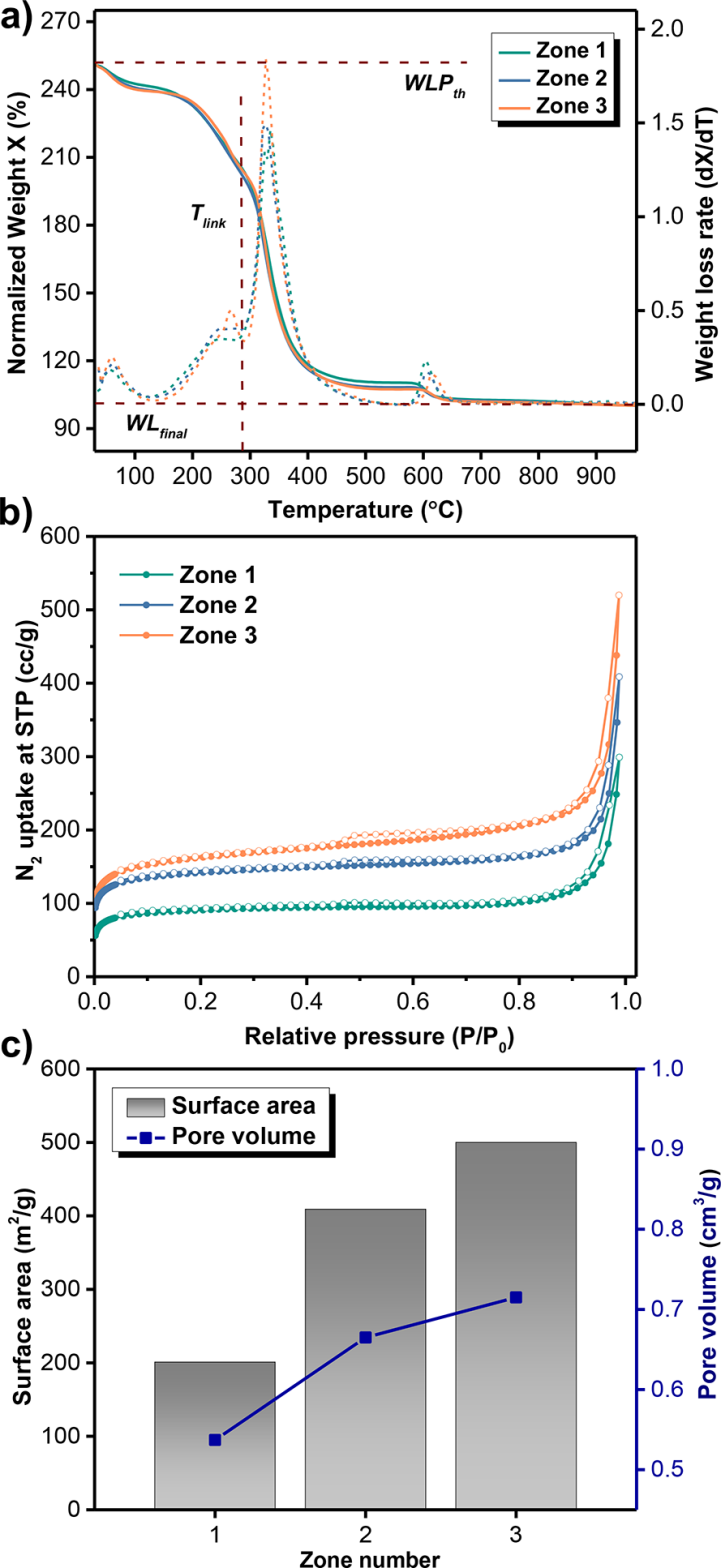
(a) TGA (left axis, solid lines) and DTG (right axis, dashed lines) curves of UiO-66(OH)_2_ particles isolated from the three reaction zones. The bottom horizontal dashed brown line represents the lower end of the theoretical TGA weight-loss plateau, WL_final_. The upper horizontal dashed brown line represents the upper end of the theoretical TGA weight-loss plateau, WLP_th_. The vertical dashed brown line represents the temperature of the combustion of the linker, T_link_. (b) Nitrogen adsorption–desorption isotherms recorded at 77 K for each zone extracted from UiO-66(OH)_2_. -●- represents adsorption, and -○- represents desorption. (c) Correlation between zone number and defects, represented by surface area and zone number of UiO-66(OH)_2_.

The thermal stability of the formed crystals was assessed via TGA analysis under continuous air flow as illustrated in Figure 2a and indicated three weight losses between 40 and 950 °C. The first weight loss visible between 40 and 150 °C was attributed to the volatilization of the residual solvents from the pores of the MOF. The second weight-loss step, which occurred between 150 and 280 °C, was assigned to the elimination of the acetic acid modulator and the dehydroxylation of the zirconium nodes. Finally, the continuous weight loss starting to appear after 280 °C is due to the decomposition of the linker which resulted in a complete decomposition of the zirconium framework and the formation of zirconium oxide (ZrO_2_) at high temperatures (>500 °C). Zirconium MOFs are widely known to contain compositional defects within their structures that were shown to be very useful in catalysis and adsorption applications.^21,39,40^ To calculate the number of missing linkers per cluster and hence deduce the number of defects for the particles formed in each zone, the TGA curves were normalized so that the final weight-loss percentage is set to be 100% and were plotted along with their first derivative (DTG) curves in Figure 2a. The number of linker deficiency was determined following the calculation method reported by Shearer et al.^39^ and is illustrated in Table S1. An increase in the number of missing linkers per Zr cluster was observed going down the tubular reactor from 1.74 in zone 1 to 1.96 in zone 3. This ascending defect trend might be a direct consequence of the reaction kinetics, where supersaturation is at its highest near the liquid–gel interface, resulting in a fast and immediate precipitation of the MOF crystals, limiting the competition between the modulator and the linker. On the other hand, and away from the interface, MOF growth dominates over nucleation, giving the modulator more time to coordinate with the zirconium clusters and hence generate more defects within the MOF structure.

**Figure 3:**
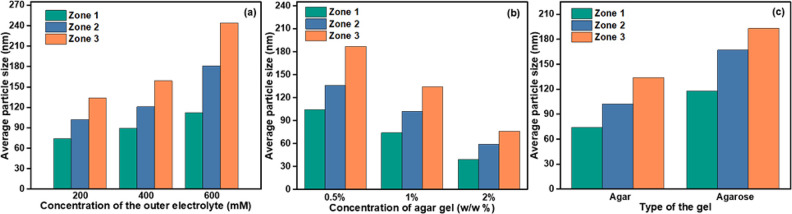
Effect of (a) concentration of the outer electrolyte, (b) concentration of the agar gel, and (c) type of gel on the particle size distribution of UiO-66(OH)_2_ particles.

It is generally agreed upon that highly defective Zr-MOFs exhibit a large BET surface area due to the greater number of missing linkers and clusters in their frameworks.^41^ The porosity and BET surface area of the nanocrystals in all zones were investigated by nitrogen adsorption/desorption experiments carried out at 77 K. As illustrated in Figure 2b, the isotherms of the Zr-MOF crystals in all zones exhibited type IV behavior with an H4 type hysteresis as opposed to the typical microporous type I isotherm commonly obtained for UiO-66 analogs synthesized via solvothermal means.^31,42^ The isotherms indicated that a significant amount of N_2_ uptake occurred at high relative pressures with a hysteresis loop closing at a relative pressure of around 0.45, suggesting a complete filling of the capillaries and the existence of both micro- and mesoporous character. This N_2_ uptake behavior indicates the formation of the hierarchical pore structure of UiO-66(OH)_2_ formed by the reaction diffusion process. Based on the sorption measurements, the BET surface area and total pore volume of the collected crystals were established and are represented in Figure 3c. Interestingly, the higher BET surface area (500 m^2^ g^–1^) and pore volume (0.72 cm^2^ g^–1^) were obtained for nanocrystals of zone 3 which is furthest from the interface, where both the defect number and average crystal size were at their highest. On the other hand, both factors were minimal near the interface in zone 1 with surface area and pore volume of 201 m^2^ g^–1^ and 0.54 cm^2^ g^–1^, respectively. This result was also confirmed by the appearance of a hysteresis loop in the zones away from liquid–gel interface (Figure 2b), suggesting the formation of mesoporous MOF crystals in zone 3 where the hysteresis was wider.^43^ For instance, while the presence of the significantly wide hysteresis loop in the isotherm of zone 3 particles indicated the mesoporous nature of the Zr-MOF in that zone, its desertion in zone 2 and disappearance in zone 1 marked the transition into a strictly microporous character.^30^ Overall, when comparing the properties of the MOF crystals prepared via our reaction–diffusion method with the ones prepared via solvothermal- and microwave-assisted means, the importance of the reaction–diffusion process in providing a synthetic tool to prepare Zr-MOF nanocrystals with controlled surface areas, pore volumes, average crystal size, and defect number at room temperature and in a one-pot synthetic route can be noted (Table S1).

### Effect of Modifying the Outer Electrolyte Concentration

After establishing the identity and characteristics of the UiO-66(OH)_2_ crystals extracted from different reaction zones, additional experiments were carried out to investigate the effect of changing the concentration of the zirconium salt, agar gel thickness, and type of the gel on the average crystal size distribution of the MOF particles (Table S2). For each reaction condition, the parameter of interest was varied, while the other experimental conditions were kept unchanged. To better compare the effect of varying these parameters on the particle size distribution, each reaction tube was divided into three equidistant zones denoted as Z1, Z2, and Z3 of the same length and position in the tube. The size of the Zr-MOF crystals in each zone (e.g., Z1 of tube 1) was first compared to the size of the crystals formed in the other two zones of the same reaction tube (Z2 and Z3 of tube 1), followed by a comparison with the average size of the crystals of the other prepared reaction tubes with different experimental conditions.

The first set of experiments, consisting of three reaction tubes, focused on varying the concentration of the zirconium salt (200, 400, and 600 mM) while keeping the concentration of the linker and agar gel percentage fixed at 20 mM and 1% w/w, respectively. As it is readily apparent in the SEM images of Figure S4, and by comparing the average particle size of the crystals obtained in the same zone number of the three reaction tubes, an increase in the MOF crystal size was observed with the increase in the concentration of the outer electrolyte from 200 mM to 600 mM. This observation can be attributed to the role of acetic acid (AA) in the system where at low concentrations of Zr(IV) salt (200 mM) a significantly smaller amount of AA can bind to the Zr(IV) clusters compared to 600 mM where relatively more clusters are available for AA to bind to. This results in a slower nucleation rate and thus the formation of larger MOF crystals.^44^ The same trend was obtained when the sizes of the crystals in different zones of the same reaction tube were compared (Figure 3a). For instance, at any given outer electrolyte concentration, the size of the crystals increases on the tubular reactor to reach 250 nm in zone 3 compared to 116 nm in zone 1 for 600 mM ZrO(NO_3_)_2_.

### Effect of Modifying the Agar Gel Concentration

In the second set, also comprising three tubular reactors, the effect of the concentration of the agar gel on the MOF average particle size distribution was investigated. This time, both the inner (ligand) and outer (metal salt) electrolyte concentrations were kept fixed at 20 and 200 mM, respectively, whereas the agar gel concentration was varied between 0.5% and 2% w/w. Comparing Z1 of each of the three tubes, the average crystal size significantly decreased when the agar gel percentage increased, suggesting an inversely proportional relationship between the agar gel concentration and the particle size distribution (Figure 3b). A similar trend was obtained in Z2 and Z3 of the reaction tubes, where larger MOF particles were produced with thinner agar gel and vice versa, as depicted in Figure 3b and Figure S5. This result was attributed to the rheological properties and texture of agar, which is known to be highly affected by the concentration and processing conditions of the gel.^45^ An increase in the concentration of agar in solution leads to a decrease in its pore size, surface area, and pore radii distribution. This results in the generation of more nucleation sites. Therefore, smaller MOF crystals are obtained as agar gel serves as a scaffold during the MOF synthesis, and its small pores will block the particle growth of the MOF.

### Effect of Modifying the Type of Gel

To investigate the effect of the gel on the average crystal size of the UiO-66(OH)_2_ particles, agarose, a polysaccharide made up of repeating D-galactose and 3,6-anhydro-L-galactose units, and a derivative of agar were employed as the gelling agents (Figure S6). Two sets of experiments were carried out at room temperature with fixed concentrations of the gel, linker, and zirconium metal (1% w/w and 20 and 200 mM, respectively) but different gel types. Comparing the size of the MOF particles isolated from the same zone number in both reaction tubes, the crystals formed in agarose exhibited a larger size compared to the ones generated in agar (Figure 3c and Figure S7). This can be explained by the fact that at a fixed gel concentration, agarose exhibits a wider pore radii distribution and hence a larger pore size compared to agar gel.^46^ This leads to an increase in the rate of MOF growth relative to nucleation as larger pores are available for the growth process of particles.

### Methylene Blue Adsorption Experiments

#### Adsorption Isotherms

To demonstrate the importance of the particle size, textural properties, and defect number of UiO-66(OH)_2_ generated via a reaction diffusion process on the adsorption capacity of the MOF in water remediation applications, UiO-66(OH)_2_ nanoparticles extracted from agar gel were employed as adsorbents for the removal of methylene blue dye from water. The performance of the MOF nanocrystals produced in our system was compared to that of a highly defective and microporous UiO-66(OH)_2_ MOF synthesized solvothermally, denoted as UiO-66(OH)_2_-Sv (Figures S8 and S9).

**Figure 4:**
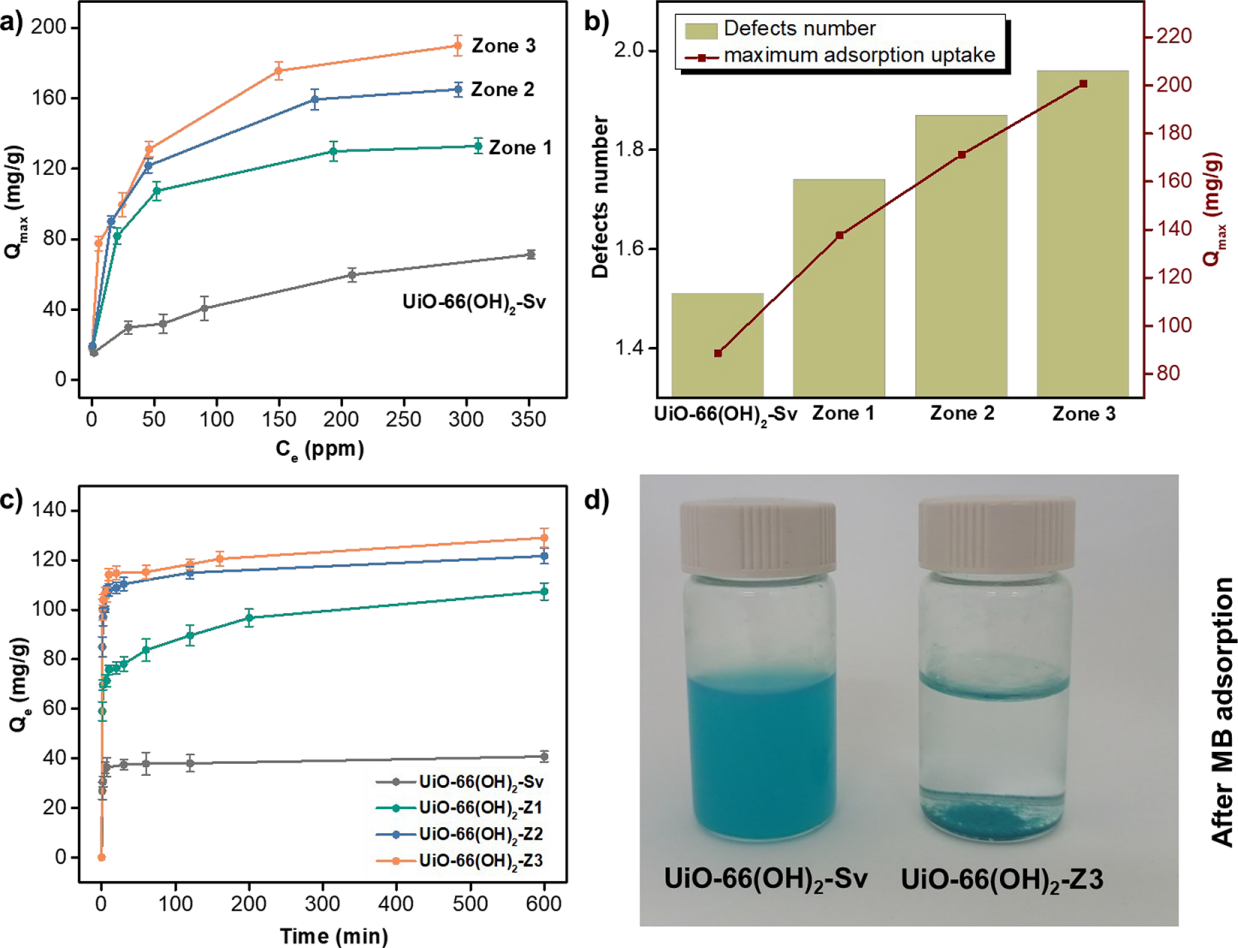
(a) The adsorption capacity of MB removal from water using UiO-66(OH)_2_-Z and UiO-66(OH)_2_-Sv at different concentrations. (b) Comparison between the defects number and the maximum adsorption capacity of UiO-66(OH)_2_-Z and UiO-66(OH)_2_-Sv. (c) 100 ppm MB dye uptake capacity onto all tested UiO-66(OH)_2_ samples as a function of contact time. (d) UiO-66(OH)_2_ samples after MB adsorption (5 min, 100 ppm) displaying the efficiency of the MOF prepared via the reaction–diffusion method in the dye removal from water.

Prior to testing the adsorption properties of UiO-66(OH)_2_ framework toward MB dye, a calibration curve was created by preparing a set of MB standard solutions with known concentrations and measuring their respective absorbances. This step was crucial since the MB supernatant following each adsorption experiment ought to be analyzed via UV–vis spectroscopy (Figure S10). The adsorption capacities of UiO-66(OH)_2_-0 particles, extracted from three different zones, toward MB at concentrations ranging between 10 and 400 ppm were evaluated (Figure 4a). As expected, the adsorption capacity, *Q*_e_, of all particles increased rapidly with an increase in the initial concentration of MB in solution before finally reaching saturation at high concentrations. This observation was justified by the fact that at low concentrations, MB was being adsorbed on the available adsorptive sites of MOFs; however, as the concentration increased, these sites became occupied and hence the maximum MB uptake was reached, resulting in a plateau. Additionally, for all initial concentrations, UiO-66(OH)_2_-Z3 had a higher adsorption capacity compared to UiO-66(OH)_2_-Z2 and UiO-66(OH)_2_-Z1, which was attributed to the increase in the number of defects, pore volume, and surface area, yielding an increase in the density of adsorption sites. More importantly, all MOFs synthesized at room temperature showed a higher adsorption capacity than UiO-66(OH)_2_-Sv due to the higher number of missing linkers per cluster that they possess as illustrated in Figure 4b.

To better understand the nature of the interaction between MB and UiO-66(OH)_2_ frameworks, the experimental adsorption isotherm data were fitted using two linear models, Langmuir and Freundlich, based on eqs 3 and 4, respectively.
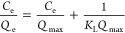
3
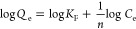
4where *Q*_max_ is the maximum quantity of MB adsorbed (in mg/g), *K*_L_ and *K*_F_ represent the Langmuir and Freundlich equilibrium constants, and the parameter *n* represents the heterogeneity of the system. The importance of these models lies in their ability to predict whether the MB contaminant is being adsorbed via a monolayer or multilayer adsorption process. For instance, the Langmuir isotherm assumes that all adsorption active sites in the MOF are equal in accessibility and that the contaminants get adsorbed on the surface of the MOF through a monolayer adsorption process, whereas the Freundlich isotherm is empirical and assumes a multilayer adsorption process that is irreversible and nonideal.^47^Figure S11 suggests that the Langmuir model depicts a better representation for the thermodynamic data for all MOFs as evidenced by the higher correlation coefficients (R^2^), determined via the least-squares regression method. This signified that the adsorption of MB onto UiO-66(OH)_2_ can be described as a monolayer adsorption process with a homogeneous surface and energy on the MOF surface. Comparing the maximum adsorption uptake (*Q*_max_) from the Langmuir data fitting for all tested UiO-66(OH)_2_ samples, UiO-66(OH)_2_-Z3 displayed the highest capacity with 203 mg g^–1^ among the synthesized MOFs in this work (Table S3, Figure 4a–d)). In addition, all the MOFs produced by the reaction diffusion process displayed higher adsorption capacity when compared to their counterparts produced solvothermally, which is probably due to their defected nature, smaller crystal size, and hierarchical micromesoporous structure. Furthermore, their performance was within the same order of magnitude when compared to the best MOF adsorbent systems reported in the literature (Table S4).

#### Effect of pH on Adsorption Capacity of UiO-66(OH)_2_

The effect of pH on the adsorption uptake was investigated for all UiO-66(OH)_2_-0 MOF samples. In brief, five different experiments were carried out by varying the pH of the initial solution which was measured to be 7.18 initially. As depicted in Figure S12, all MOF samples exhibited an increase in the sorption affinity toward MB when the pH increased from 3.5 to 11. In addition, MB adsorption uptake increased on going from the lowest defected framework, UiO-66(OH)_2_-Sv, to the highly defected UiO-66(OH)_2_-Z3. Based on these results, we reasoned that besides the large surface area of UiO-66(OH)_2_-Z3 which is enhancing the removal uptake of MB, electrostatic interactions between the dye and the framework might be taking place. Zeta-potential measurements of the best performing MOF, that is, UiO-66(OH)_2_-Z3, revealed a zero-point charge at 3.5, with a positive charge of the framework surface below this pH and a negative charge above it (Figure S13). With MB being a positively charged dye, our results suggested strong electrostatic interactions between the framework and the dye at pHs higher than the zero-point charge due the interactions between negatively charged surface of the MOF and the positively charged dye. On the other hand, the lowest uptake capacity achieved at a pH of ∼3.5 was attributed to the positively charged nature of both the adsorbent and adsorbate which limited the capacity of electrostatic attractions and therefore ion uptake. Furthermore, and under acidic conditions, the free hydroxyl groups attached to the ligand exist in the −OH/–OH_2_^+^ state. Hence, methylene blue, which is positively charged in water, will attach to the MOF surface and pores via ion exchange and weak physical interactions, which explains the low sorption uptake. On the other hand, and under basic conditions, the hydroxyl group gets deprotonated, and thus, MB gets adsorbed through electrostatic interactions. Although some −OH groups might remain present in basic conditions, the weak interactions between these groups and MB are masked by the stronger electrostatic forces between the latter and the deprotonated hydroxyl groups.^48^

#### Adsorption Kinetics

To explore the mechanistic aspects of the adsorption of MB ions onto UiO-66(OH)_2_, the uptake capacity as a function of the contact time was studied for an initial concentration of 100 ppm. Closely examining the kinetic curves in Figure 4c, we found the adsorption rate to undergo two distinct phases. The first phase, accounting for the first 7 min of contact time between the MOF adsorbents and MB was fast and guided by the high dye concentration gradient in the solution. The adsorption rate, however, slowed down eventually as the adsorbing sites became occupied by the MB ions to finally reach a plateau after 10 min. In terms of efficiency, MB adsorption by UiO-66(OH)_2_-Z3 appeared to outperform its counterparts extracted from zones 1 and 2 in addition to the frameworks prepared via a solvothermal process (Figure 4d).

To determine the mode of interactions between the framework materials and MB, the experimental data were fitted into two kinetic models, a pseudo-first-order model (eq 5) and pseudo-second-order model (eq 6).^49^ These models help in determining the type of interactions between the dye and the frameworks.
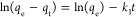
5
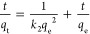
6where *q*_e_ (mg g^–1^) is the equilibrium adsorption capacity, *q*_t_ (mg g^–1^) is the adsorption quantity at time *t* (min), and *k*_1_ (min^–1^) is the pseudo-first-order rate constant and *k*_2_ (g mg^–1^ min^–1^) the pseudo-second-order rate constant.

The fitting data in Figure S14 suggested a bimolecular adsorption process, whereby the rate-determining step depends on the MOF nanocrystals and the dye concentrations. This was based on the high regression coefficient value (*R*^2^) obtained in the case of the pseudo-second-order model for all UiO-66(OH)_2_ MOFs. This result suggested the formation of strong bonds between the adsorption sites of the MOFs (e.g., defect sites of the MOFs and free hydroxyl groups attached to the linkers) and the positive dyes. To acquire a better analysis of the adsorption mechanism, the intraparticle diffusion model developed by Weber and Morris was examined by plotting *q*_t_, previously defined, versus the square root of time as shown in eq 7.
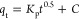
7where *K*_p_ is the intraparticle diffusion rate constant, and *C* is a constant defining the boundary layer effect. As depicted in Figure S15, two linear portions are observed for each plot, indicating a two-stage diffusion process. This multilinearity suggested that an external surface adsorption was taking place at the beginning of the adsorption experiment where MB was rapidly binding to the defected surface and diffusing onto the large pores of the MOFs as indicated by the steep slope in the first stage. This was followed by a progressive adsorption into the pores and binding sites of the frameworks until equilibrium is finally reached, resulting in a controlled intraparticle diffusion phenomenon. This second stage depicts the intraparticle diffusion mode which directly depends on the thickness of the boundary layer effect, portrayed by parameter *C* in eq 7, and is considered the rate-controlling step when *C* is zero.^50^ To determine the rate-limiting step, linear fits of the second portion of each curve were characterized by their slope and intercept. As the extrapolation of the linear fits did not pass through the origin in all cases, it could be inferred that intraparticle diffusion is not the rate-determining step, and more than one adsorption process is affecting the MB adsorption in this case. Furthermore, a direct comparison between the plots of UiO-66(OH)_2_-Sv and the three zones of UiO-66(OH)_2_ indicated a higher *q*_t_ for the latter at each time interval *t*^0.5^, thus proving the enhanced adsorption capacity of MOFs synthesized via the reaction–diffusion process.

#### Recyclability of the MOF

The ability to recycle and reuse MOF materials after ion capture makes them unique compared to other conventional adsorbents.^51,52^ An adsorbent that maintains high adsorbing efficiency after several adsorption cycles is highly desirable, cost effective, and practical for industrial and commercial applications. For this aim, the highly efficient postadsorbing UiO-66(OH)_2_-Z3 was dispersed in methanol followed by sonication for 1 h and finally activation at 100 °C overnight. Following three consecutive MB removal cycles, no significant change in the adsorption efficiency was detected at 15 ppm, as displayed in Figure S16, signaling the high efficiency and stability of the MOF in dye adsorption. Moreover, PXRD, SEM, and BET measurements of all UiO-66(OH)_2_-0 samples were performed following one cycle of adsorption to gain insight into the properties of the frameworks after adsorption. PXRD and SEM analyses indicated the stability of the frameworks where no changes in morphology nor in structural properties were seen after adsorption, as evidenced by the absence of any additional diffraction patterns arising in UiO-66(OH)_2_ after MB capture (Figures S17 and S18) Furthermore, BET analysis revealed a significant decrease in the surface area of all UiO-66(OH)_2_ MOFs prepared in the agar gel, suggesting the blockage of the pores of the material after MB adsorption (Table S5).

## 4 Conclusion

In summary, UiO-66(OH)_2_ particles with tuned crystal size distribution and defect levels were prepared via a reaction–diffusion process at room temperature. The hierarchical porous crystals, formed at the bottom of the tubular reactor, exhibited a surface area up to 500 m^2^ g^–1^ and defect number of 1.96 compared to the crystals formed at the liquid–gel interface having a surface area of 201 m^2^ g^–1^ and defect number of 1.74. The synthetic chemical parameters of these systems, i.e., the concentrations of the zirconium salt and agar gel, in addition to the type of gelling agent were found to govern the morphological and physical properties of the obtained crystals. Methylene blue dye removal was examined to assess the influence of crystal size, porosity, and surface area on the adsorptive properties of UiO-66(OH)_2_. The crystals that formed farthest from the interface exhibited a removal efficiency of 202 mg/g compared to the crystals that formed at the interface with an uptake capacity of 137 mg/g. The adsorption and kinetic studies revealed a strong interaction between the framework and the dye which was being adsorbed via a monolayer process inside the hierarchical micromesoporous structure. Compared to the conventional solvothermal method used to generate MOFs, the reaction–diffusion approach yielded MOFs with tuned chemical features and enhanced adsorptive performance, thus demonstrating the potential of energy efficient synthesis procedures for the generation of additional MOF materials for industrial and commercial applications.

## Supporting Information Available

Experimental methods, physical and chemical properties, effects of chemical parameters on the crystal size of UiO-66(OH)_2_, adsorption isotherms, kinetic isotherms, and MOF regeneration experiments (PDF)
