# Supplementary material for: Controlled Growth of Highly Defected Zirconium–Metal–Organic Frameworks via a Reaction–Diffusion System for Water Remediation
Source: ACS Appl Mater Interfaces. 2024 Jan 17;17(12):17741–50. doi: 10.1021/acsami.3c16327 (PMC11955948; doi:10.1021/acsami.3c16327)
Supplement: Supplementary file 1 — am3c16327_si_001.pdf [file am3c16327_si_001.pdf]

Supporting Information

Controlled Growth of Highly Defected Zirconium-Metal-Organic Frameworks via a Reaction-Diffusion System for Water Remediation

Patrick Damacet,[a,b] Karen Hannouche,[a] Abdelaziz Gouda,[c] Mohamad Hmadeh*,[a]

a. Department of Chemistry, Faculty of Arts and Sciences, American University of Beirut, Beirut 1107 2020, Lebanon
b. Department of Chemistry, Burke Laboratory, Dartmouth College, Hanover, New Hampshire 03755, United States
c. Department of Chemistry, University of Toronto, 80 St. George Street, M5S 3H6, Toronto, Canada

Email: Mohamad.Hmadeh@aub.edu.lb

Table of Contents

1. Materials and methods 2
1.1. Materials 2
1.2. Synthesis method 2
1.3. Characterization techniques 3
2. Conventional reaction-diffusion system for UiO-66(OH)$_2$ preparation 4
3. Chemical and structural properties of the framework 4
4. Effect of varying the chemical parameters on the average particle size distribution 7
4.1. Effect of varying the concentration of the outer electrolyte 7
4.2. Effect of varying the concentration of the agar gel 8
4.3. Effect of varying the type of gel 9
5. Synthesis and characterization of UiO-66(OH)2-Sv 10
5.1. Solvothermal synthesis procedure of UiO-66(OH)$_2$-Sv 10
5.2. Structural and chemical properties of UiO-66(OH)$_2$-Sv 10
6. Methylene blue dye uptake studies 12
6.1. Calibration curve for MB 12
6.2. Adsorption isotherms 13
6.3. Effect of pH on the adsorption capacity of the framework 15
6.4. Kinetic isotherms 16

6.5. Intra-particle diffusion model 17
6.6. Recyclability and stability of UiO-66(OH)$_2$ after dye adsorption 17
7. References 19

1. Materials and methods

1.1. Materials

All chemicals used in this research work were commercially available and directly utilized as received without additional purification. 2,5-dihydroxyterephthalic acid ($C_8H_6O_6$, 99%) and methylene blue ($C_{16}H_{18}ClN_3S.xH_2O$) were purchased from Sigma-Aldrich. Zirconyl nitrate hydrate ($(ZrO(NO_3)_2.xH_2O$, 99.5%, 4% Hf), acetic acid (CH_3COOH, >99%), and *N,N*-dimethylformamide (DMF, Analytical reagent grade) were purchased from Thermo Scientific. Agarose ($C_{12}H_{18}O_9$, molecular biology grade) was acquired from Fisher bioreagents, and finally Bacteriological agar gel ($(C_{12}H_{18}O_9)_n$) was obtained from Biolab Zrt.

1.2. Synthesis method

UiO-66(OH)$_2$ was prepared via a continuous precipitation reaction at room temperature in a gelled medium. The inner electrolyte was prepared by dissolving 118 mg of 2,5-dihydroxyterephthalic acid (20 mM) in 13 mL *N*,*N*-dimethylformamide (DMF) followed by heating for 3 mins to ensure complete dissolution of the linker. After the color of the mixture changed from yellow to orange red, 13 mL of deionized water (DI) were added to the solution along with 300 mg (1% w/w) of agar powder. The mixture was then stirred and heated over a hot plate for a few minutes until the agar gel dissolved completely. Following this, 4 mL of acetic acid (acting as a modulator) were added, and the obtained mixture was poured into a Pyrex test tube filling its two third. Finally, the test tube was covered with parafilm for 2 hours to allow complete gelation of agar matrix before adding the outer electrolyte.

The outer solution was prepared by dissolving 550 mg of zirconyl nitrate hydrate (200 mM) in a 12 mL solution made of a 9:1 mixture of DI/DMF. After sonication at room temperature, the obtained solution was poured on top of the inner electrolyte and the test tube was covered with parafilm for several days to propagate. Eventually, the precipitation regions of UiO-66(OH)$_2$ crystals were divided into three equidistant consecutive zones of 1 cm thickness and extracted into different falcon conical tubes. The crystals in each zone were vigorously washed with hot deionized water to dissolve the agar gel and later activated by solvent exchange with ethanol.

Finally, the particles were collected by centrifugation and dried under vacuum at 110°C for 12 hours.

1.3. Characterization techniques

A variety of characterization techniques were utilized to gain a detailed description of the properties and characteristics of the synthesized UiO-66$(OH)_2$ samples. Powder X-ray diffraction (PXRD) patterns of all samples were recorded using a Bruker D8 advance X-ray diffractometer with a Cu Kα radiation (k=1.5418 Å), a 40 kV voltage, and a 40 mA current, in which the 2θ range was between 5° and 30°, at an increment of 0.02° (Bruker AXS GmbH, Karlsruhe, Germany). The thermal stabilities of the synthesized samples were investigated via thermogravimetric analysis (TGA 209 Libra from Netzsch) where a mass of 5 mg of each MOF from each zone was weighed using a microbalance and placed in an 85µl alumina crucible (6.8 mm x 4 mm). The crucible was then placed in the auto-sampler which places the sample in the oven where it is heated in the presence of nitrogen and air from 30 °C to 1000 °C at a 10 $°C.min^{-1}$ heating rate. The morphologies and sizes of the crystals for all samples were evaluated using scanning electron microscopy (TESCAN MIRA3 SEM coupled with EDX). For SEM imaging, a very small amount of the MOF was placed on a conductive carbon tape covering an aluminum SEM specimen stub, which was then coated with a thin layer of gold (around 15 nm) and placed in the electron microscope for imaging at a low beam voltage of 5 kV to prevent charging and edge effects. Furthermore, Nitrogen sorption measurements were performed at 77 K for each zone in UiO-66$(OH)_2$-0 to assess their surface area and pore volume using Micrometrics 3Flex surface characterization analyzer, in which the samples were degassed under a nitrogen flow at 125°C overnight prior to the analysis. Finally, the UV-Vis spectrophotometric measurements were carried out on a JASCO V-550 UV/Vis spectrophotometer using WPI Quartz Cuvettes (12.5 x 12.5 x 45 mm) with a band width of 10 mm and a volume of 3.5 mL.

2. Conventional reaction-diffusion system for UiO-66(OH)$_2$ preparation

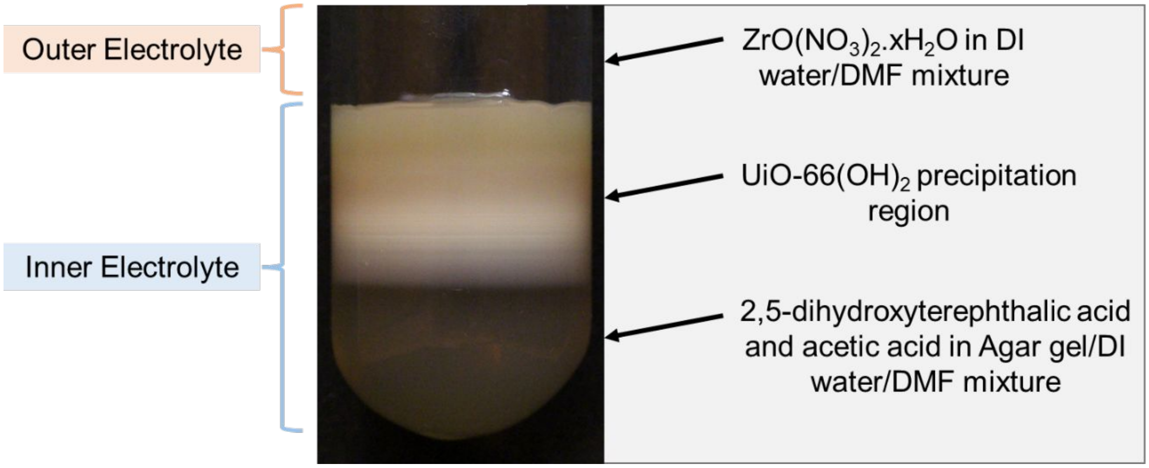

Figure S1. Experimental setup of the generation of UiO-66(OH)$_2$ particles via a reaction-diffusion process. Zr(IV) salt diffuses into an agar gel matrix made up of the ligand and the modulator to form MOF particles with different sizes.

3. Chemical and structural properties of the framework

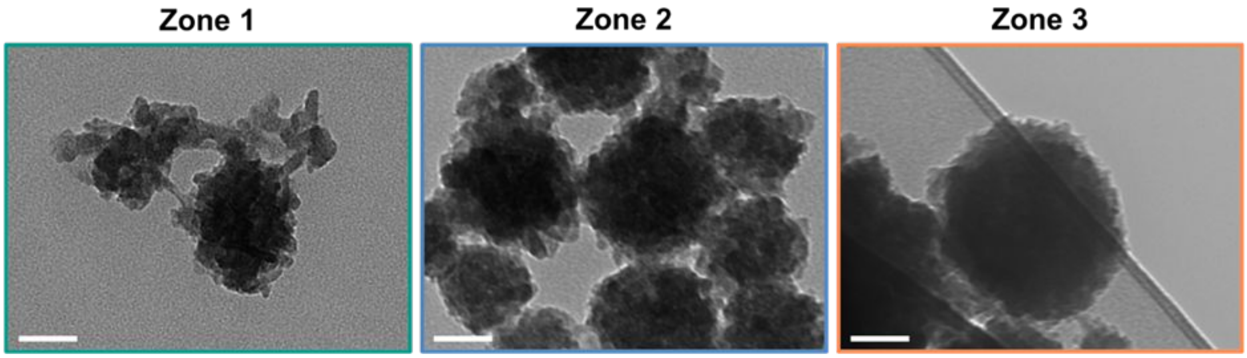

Figure S2. TEM images of the MOF particles extracted from the three reaction zones. Note SEM scale bar is 50 nm.

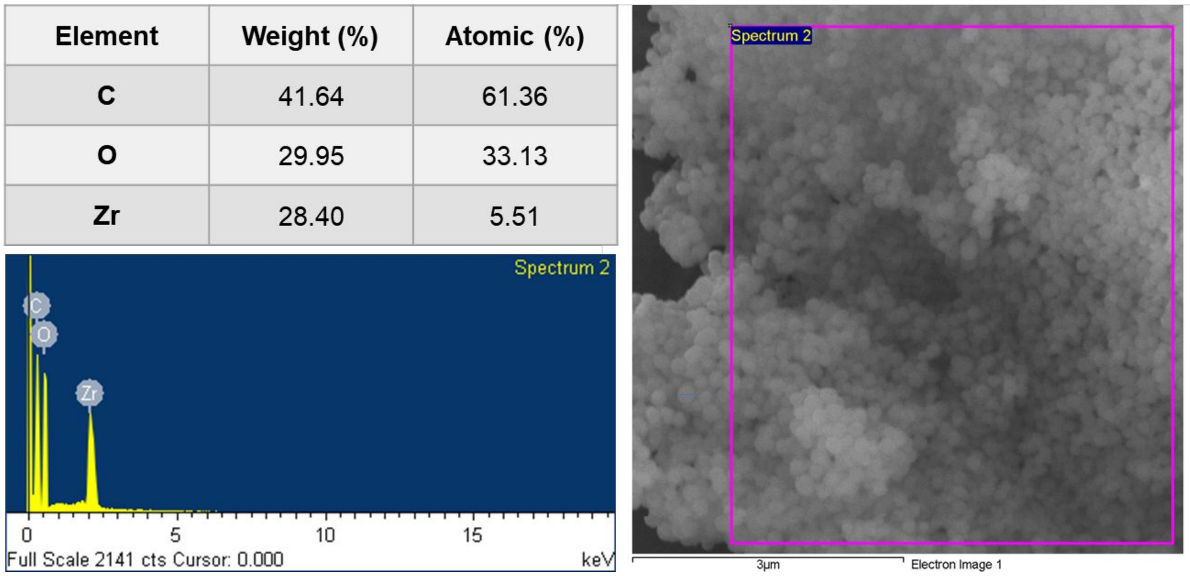

Element	Weight (%)	Atomic (%)
C	41.64	61.36
O	29.95	33.13
Zr	28.40	5.51

Figure S3. SEM and EDX analysis of UiO-66$(OH)_2$ extracted from zone 3 of the tubular reactor.

MOF Sample	Particle size[a] (nm)	Particle size[b] (nm)	BET surface area (m^2/g)	Pore Volume (cm^3/g)	Defects number	Ref
**UiO-66$(OH)_2$-Z1**	74	58	201	0.54	1.74	This work
**UiO-66$(OH)_2$-Z2**	102	91	409	0.66	1.87	This work
**UiO-66$(OH)_2$-Z3**	134	119	500	0.72	1.96	This work
**UiO-66$(OH)_2$-Sv1**	252	-	602	0.19	1.51	[1]
**UiO-66$(OH)_2$-Sv2**	100	29.7	567	0.46	-	[2]
**UiO-66$(OH)_2$-Sv3**	31.9	25.6	222	-	-	[3]

Table S1. Quantitative results extracted from the characterization performed on each UiO-66$(OH)_2$-Z along with UiO-66$(OH)_2$-Sv synthesized via solvothermal means and reported in the literature. Particle size "a" is estimated using SEM and Particle size "b" is calculated using the Scherrer equation from the PXRD diffraction peaks.

	Experimental parameters studied			
	**Sample name**	**Concentration of Metal cation (mM)**	**Concentration of gel**	**Type of gel**
**Reference**	UiO-66$(OH)_2$-0	200	1% w/w	Agar gel
**Effect of outer electrolyte concentration**	UiO-66$(OH)_2$-1	400	1% w/w	Agar gel
	UiO-66$(OH)_2$-2	600	1% w/w	Agar gel
**Effect of the thickness of the agar gel**	UiO-66$(OH)_2$-3	200	0.5% w/w	Agar gel
	UiO-66$(OH)_2$-4	200	2% w/w	Agar gel
**Effect of the type of gel used**	UiO-66$(OH)_2$-5	200	1% w/w	Agarose

Table S2. Synthetic conditions used for the generation of UiO-66$(OH)_2$ frameworks throughout this study.

4. Effect of varying the chemical parameters on the average particle size distribution

4.1. Effect of varying the concentration of the outer electrolyte

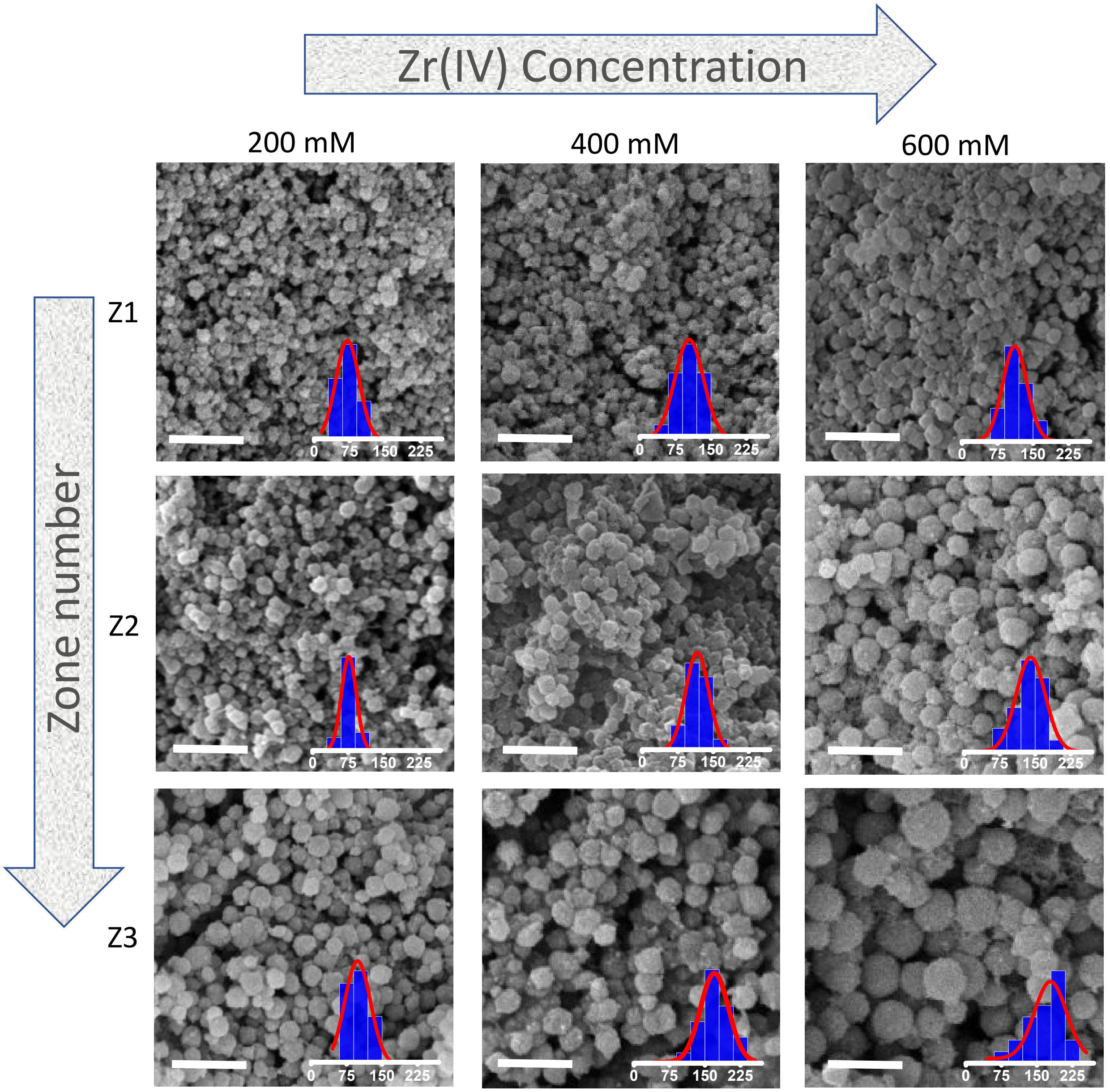

Figure S4. Scanning electron microscope images showing the growth patterns of UiO-66$(OH)_2$-0, UiO-66$(OH)_2$-1, and UiO-66$(OH)_2$-2 crystals at different outer electrolyte concentrations in all reaction zones. Note the scale bar is 500 nm.

4.2. Effect of varying the concentration of the agar gel

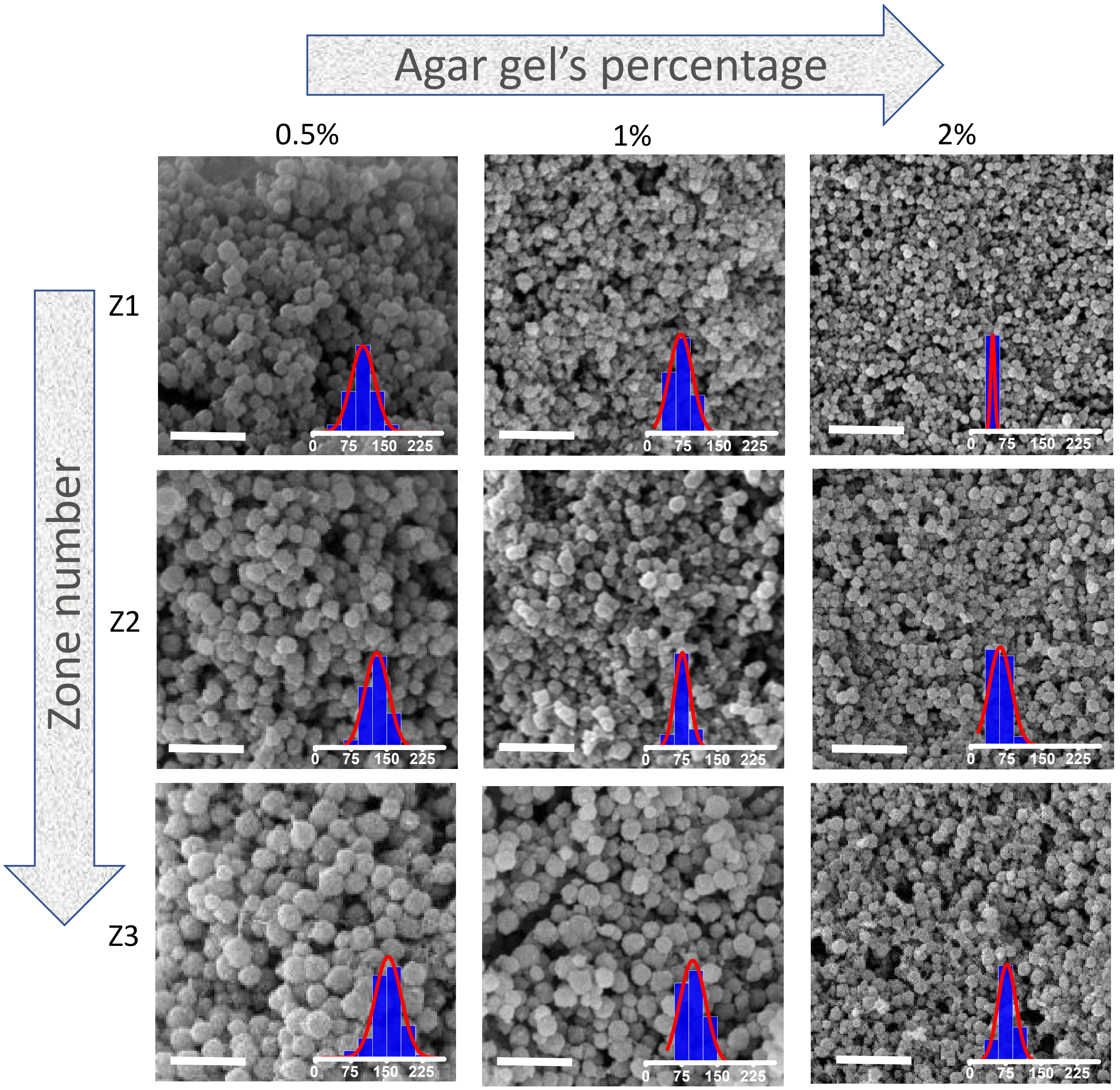

Figure S5. Scanning electron microscope images showing the growth patterns of UiO-66(OH)$_2$-0, UiO-66(OH)$_2$-3, and UiO-66(OH)$_2$-4 crystals at different concentrations of agar gel in all reaction zones. Note the scale bar is 500 nm.

4.3. Effect of varying the type of gel

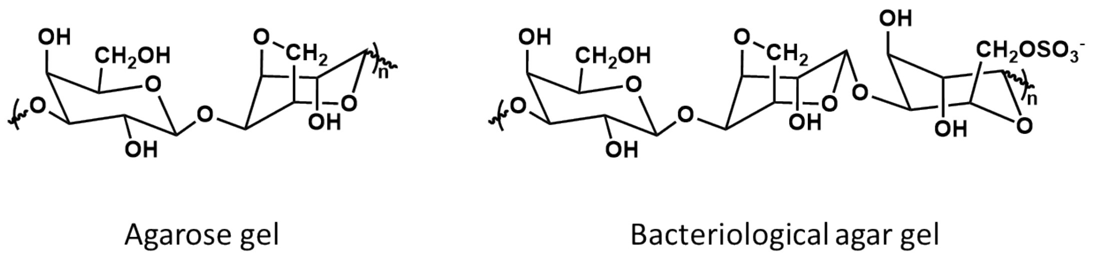

Figure S6. Chemical structures of Agarose and bacteriological agar gels.

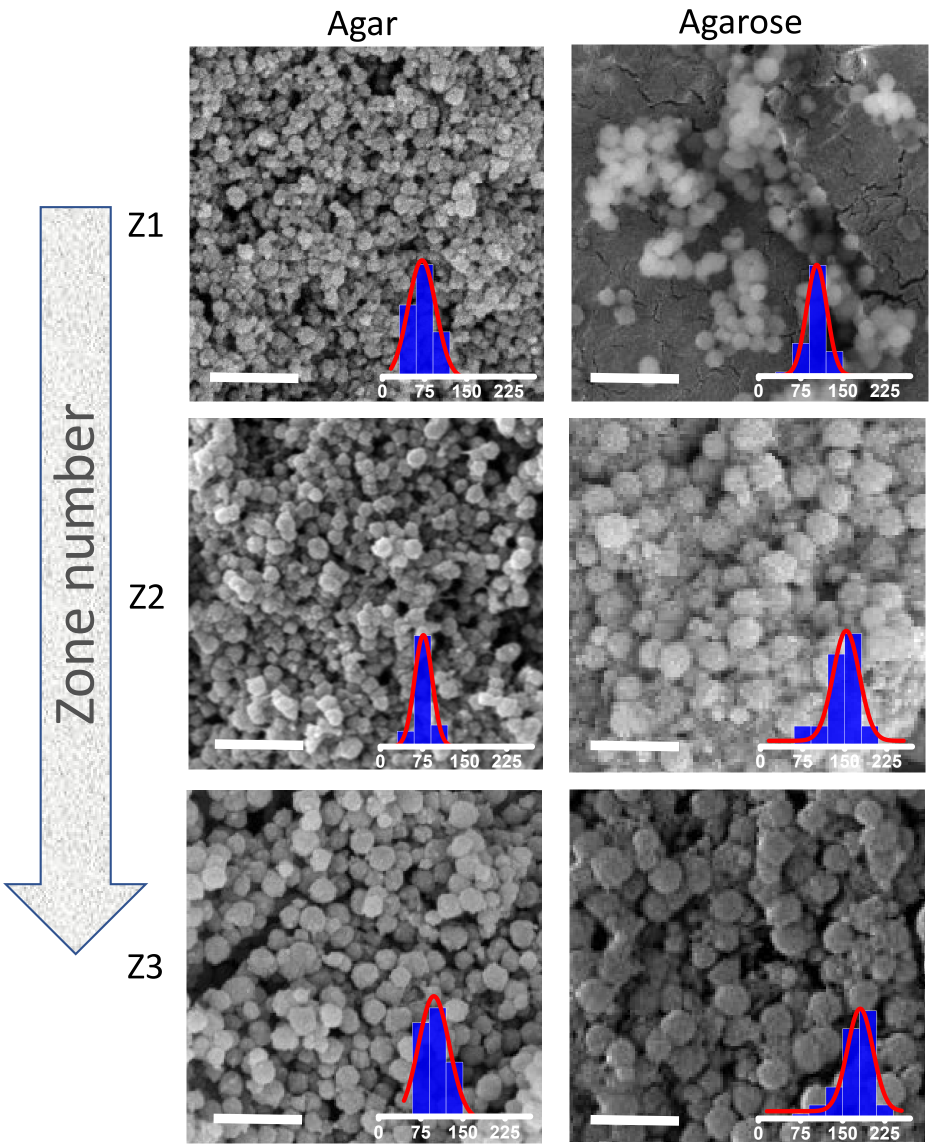

Figure S7. Scanning electron microscope images showing the growth patterns of UiO-66$(OH)_2$-0 and UiO-66$(OH)_2$-5 crystals using different solidifying agents in all reaction zones. Note the scale bar is 500 nm.

5. Synthesis and characterization of UiO-66(OH)2-Sv

5.1. Solvothermal synthesis procedure of UiO-66(OH)$_2$-Sv

UiO-66(OH)$_2$-Sv was synthesized via a solvothermal method. In brief, equimolar amounts of 2,5-dihydroxyterephthlalic acid (1.48 mmol, 293 mg) and $ZrCl_4$ (1.48 mmol, 344 mg) were dissolved in 100 mL reactive vial containing 40 mL of DMF. After sonicating the mixture for a few minutes to allow for the complete dissolution of the MOF's reagent, 11 mL of formic acid were added dropwise to the reaction mixture which was further sonicated for 5 minutes. The reactive vial was then placed in a preheated oven set at 120°C for 21 hours before being allowed to cool down to room temperature. The resulting white powder was isolated by centrifugation and vigorously washed with 300 mL DMF (6 x 50 mL) to dissolve all unreacted reagents. The obtained MOF was then activated via solvent exchange with acetone (4 x 50 mL) and placed in a vacuum oven set at 85 °C for 12 hours.

5.2. Structural and chemical properties of UiO-66(OH)$_2$-Sv

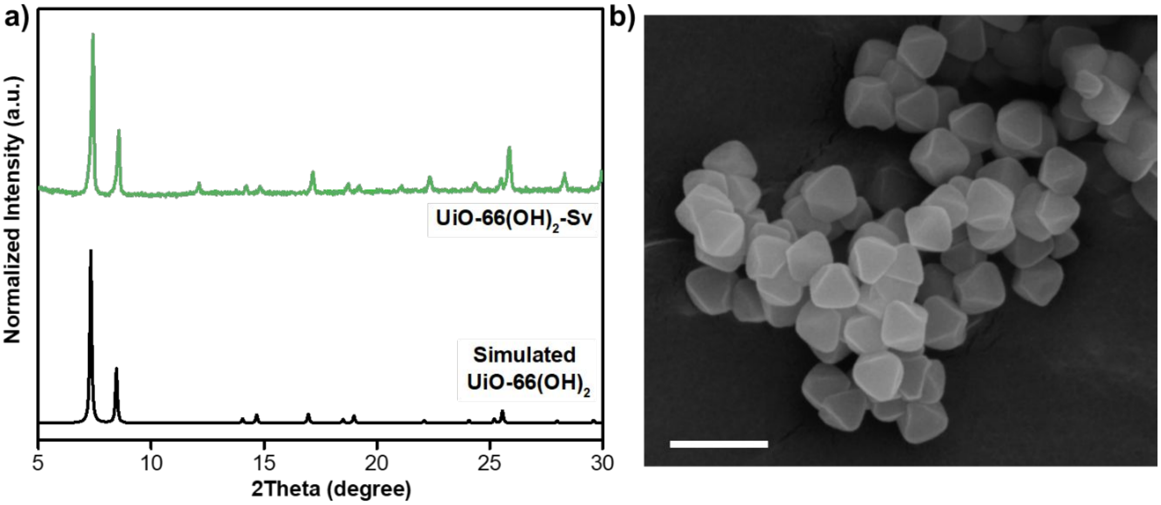

Figure S8. PXRD and SEM analysis of UiO-66(OH)$_2$-Sv. Note the SEM scale bar is 500 nm.

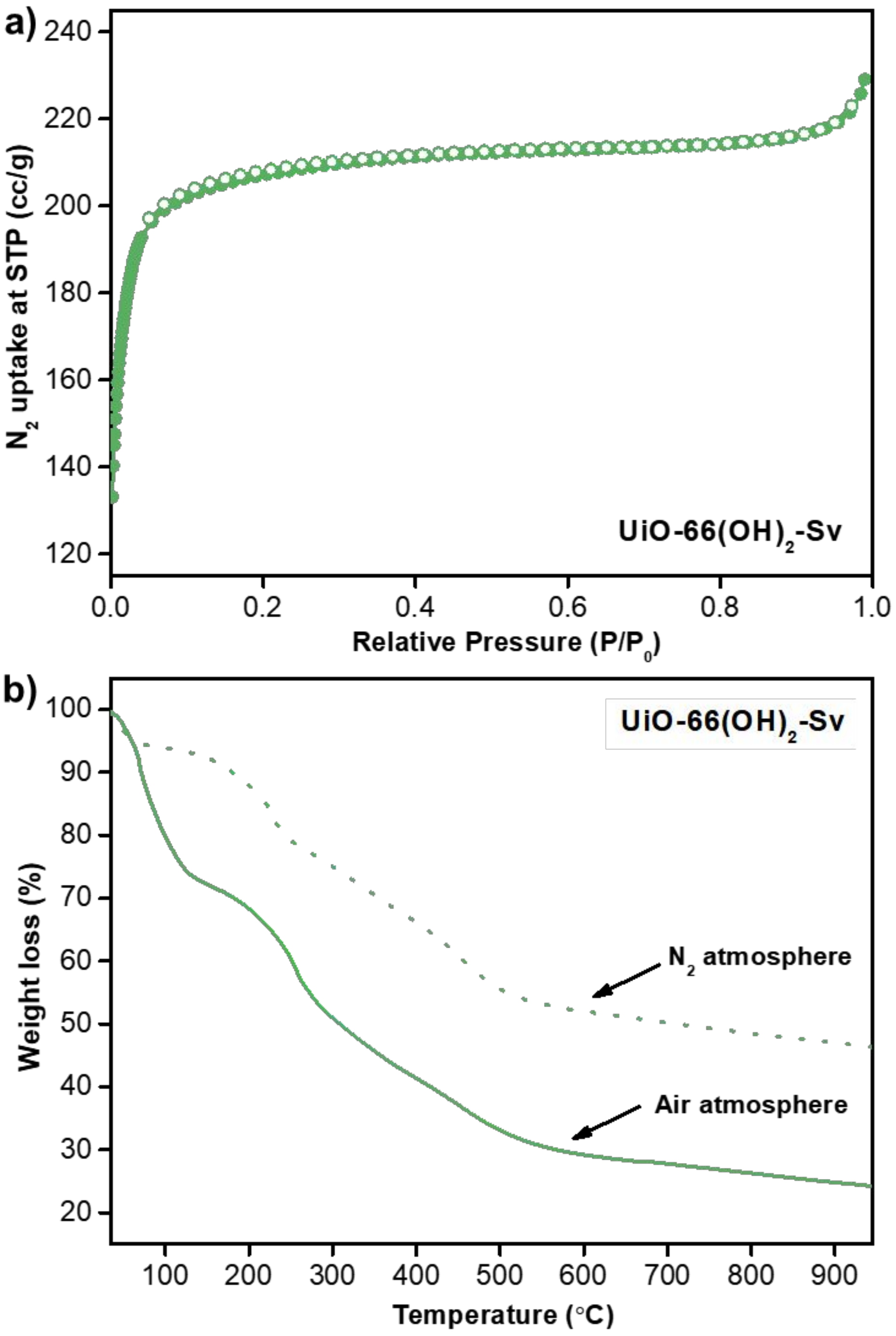

Figure S9. (a) N_2 adsorption/desorption isotherms data and (b) TGA curves of UiO-66(OH)$_2$-Sv nitrogen and air atmospheres.

6. Methylene blue dye uptake studies

6.1. Calibration curve for MB

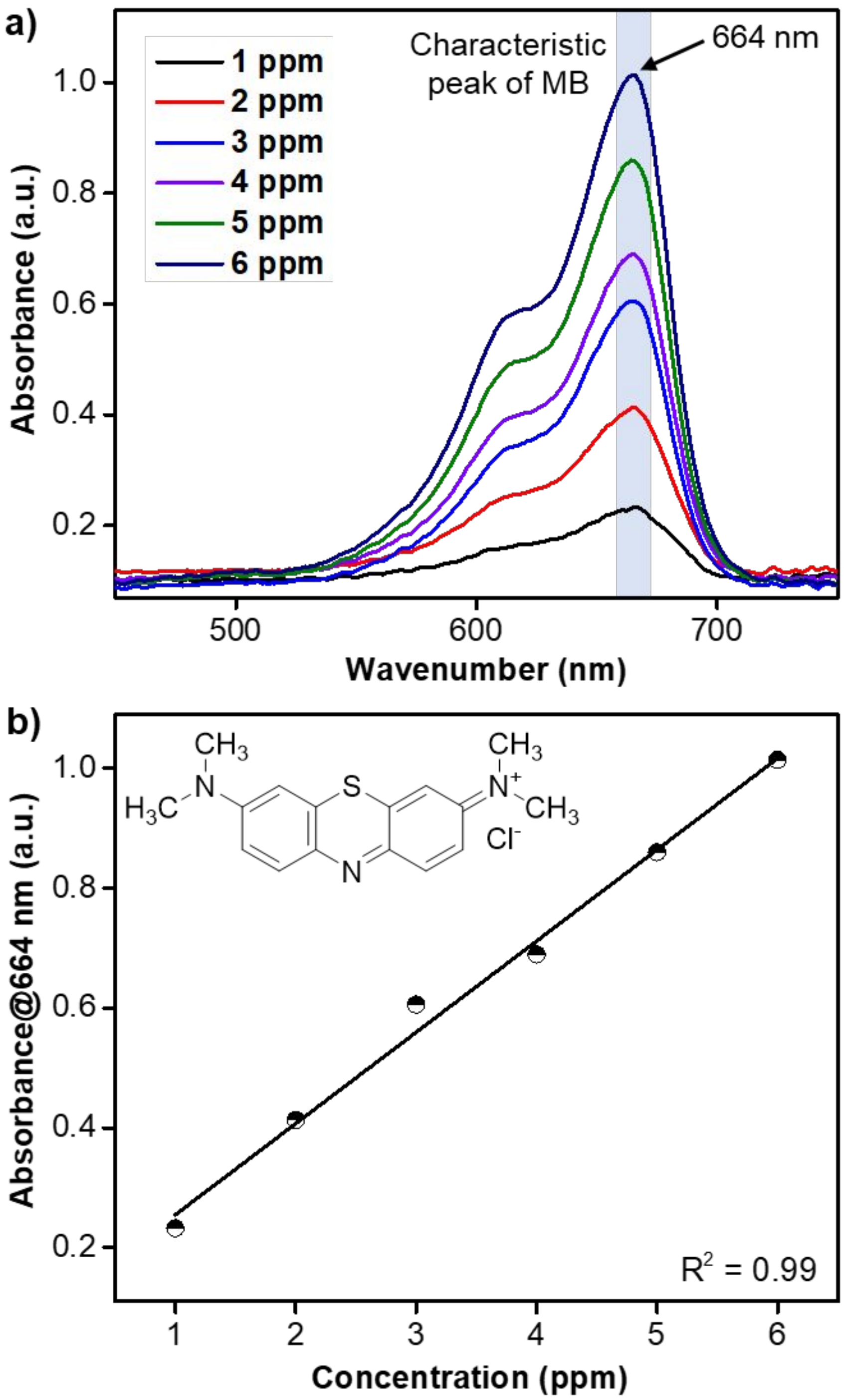

Figure S10. a) UV-Vis absorption spectra of Methylene Blue (MB) at different concentrations and b) Calibration curve of MB (inset: Chemical structure of MB).

6.2. Adsorption isotherms

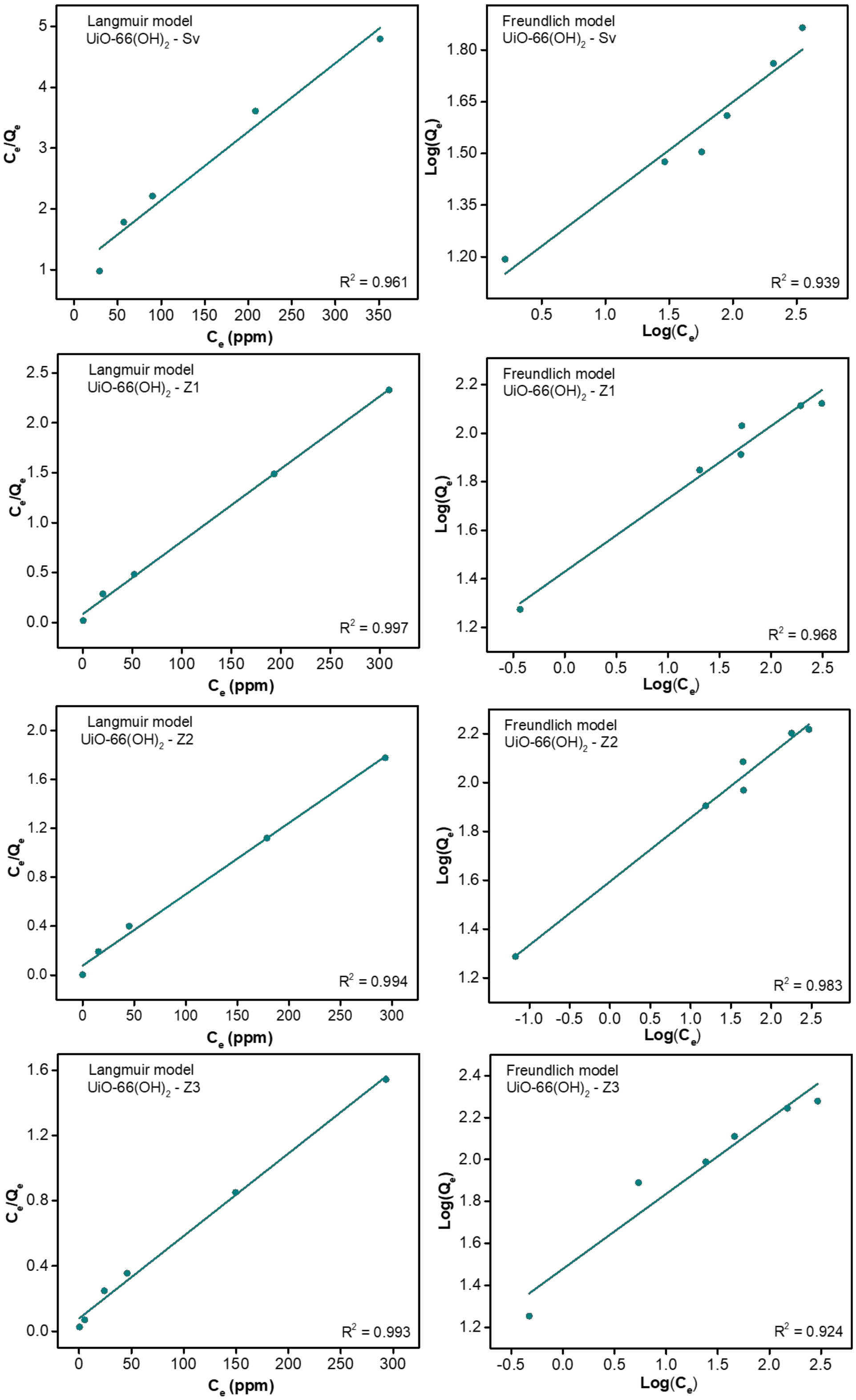

Figure S11. Adsorption data fitting using both Langmuir and Freundlich linear models for UiO-66(OH)$_2$-Sv and UiO-66(OH)$_2$-0 tested samples.

Sample	Q_{max} (mg/g)
**UiO-66(OH)$_2$- Z1**	137
**UiO-66(OH)$_2$- Z2**	171
**UiO-66(OH)$_2$- Z3**	201
**UiO-66(OH)$_2$- Sv**	88

Table S3. The maximum adsorption uptake for all tested MOFs extracted from the Langmuir data fitting given in Equation 3.

Adsorbent	Q_{max} (mg/g)	Ref
**UiO-66/MIL-101(Fe)-GOCOOH composite**	448.71	4
**UiO-66(NH$_2$)**	91	5
**MOF-199**	84	6
**OH-MOF-199 (1:1)**	263	6
**OH-MOF-199 (3:1)**	147	6
**OH-MOF-199 (1:3)**	111	6
**Cu-BTC**	40	7
**Fe_3O_4@SiO_2@Zn–TDPAT**	58	8
**MOF-199/GO**	183	9
**Cd-MOF**	149	10
**Fe_3O_4/ $Cu_3(BTC)_2$**	244	11
**MIL-101 (A$_3$)**	11	12
**PAN/ZIF-8 nanofiber membranes**	224	13
**Co doped Fe-BDC**	24	14
**UiO-66(OH)$_2$ - Z3**	201	**This Work**

Table S4. Maximum uptake capacity of UiO-66(OH)$_2$- Z3 compared with other MOF systems reported in literature.

6.3. Effect of pH on the adsorption capacity of the framework

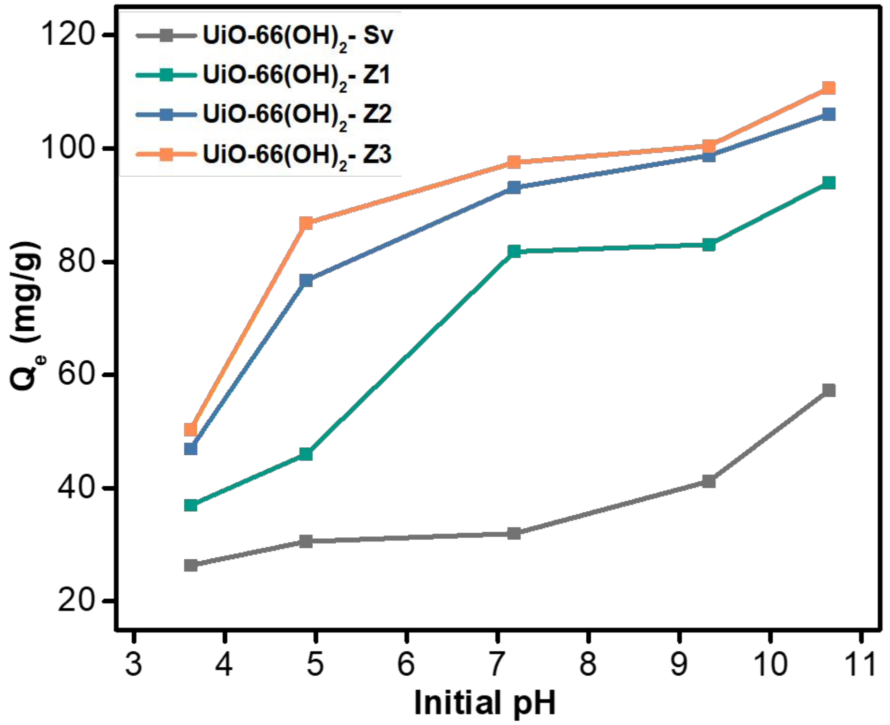

Figure S12. Adsorption uptake of MB with all tested MOFs at different pH values for an initial concentration C_0 of 80 ppm.

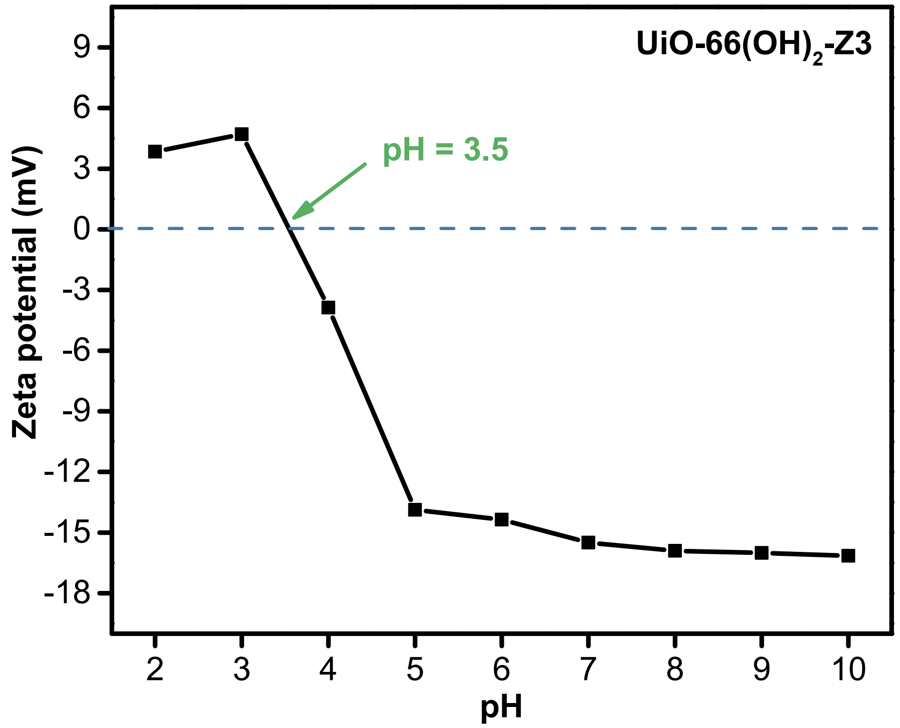

Figure S13. Zeta potential measurements for UiO-66$(OH)_2$-Z3 at different pHs.

6.4. Kinetic isotherms

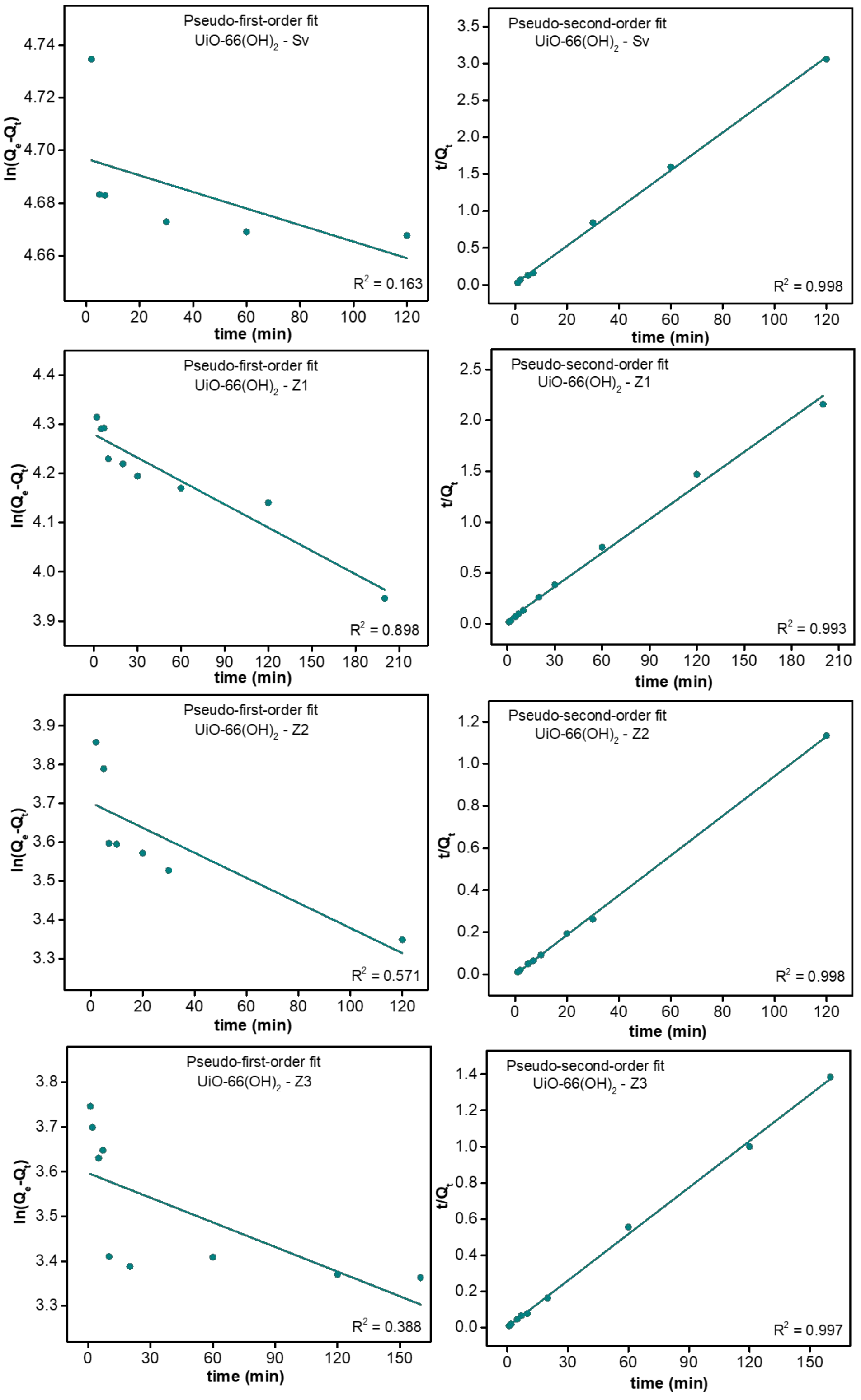

Figure S14. Kinetic data fitting using both pseudo-first and pseudo-second order models for UiO-66(OH)$_2$-Sv and UiO-66(OH)$_2$-0 samples.

6.5. Intra-particle diffusion model

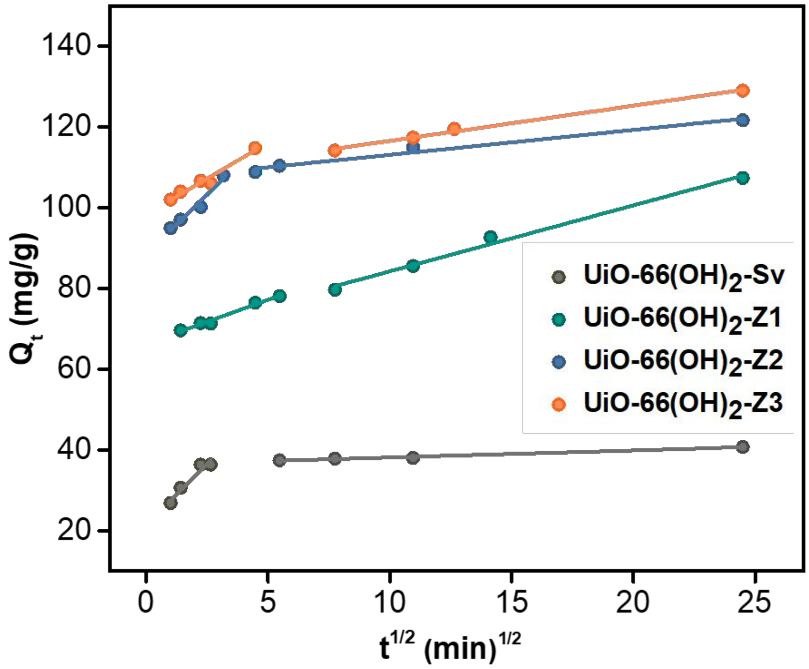

Figure S15. Intraparticle diffusion model for MB adsorption onto the different UiO-66(OH)$_2$-0 and UiO-66(OH)$_2$-Sv samples.

6.6. Recyclability and stability of UiO-66(OH)$_2$ after dye adsorption

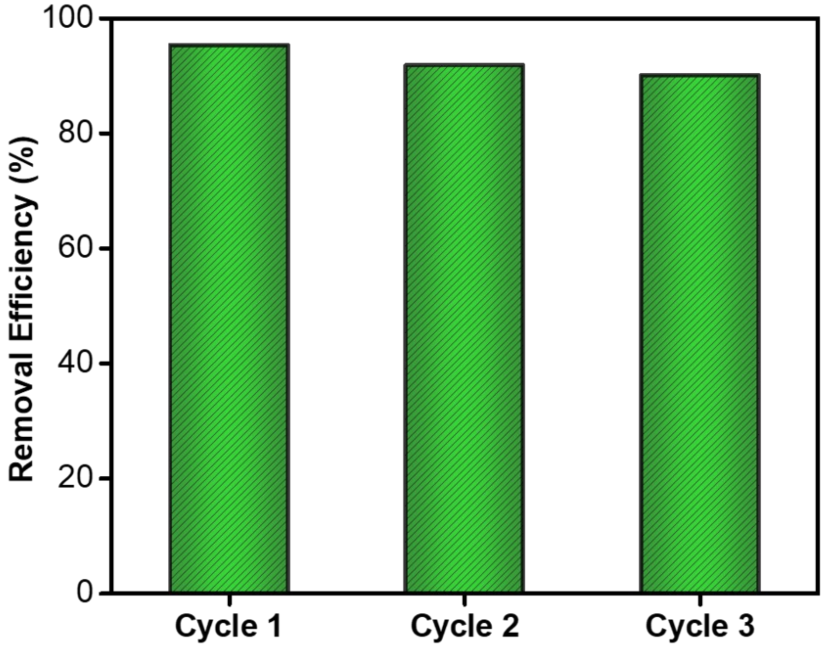

Figure S16. Reusability of UiO-66(OH)$_2$- Z3 for MB removal from water.

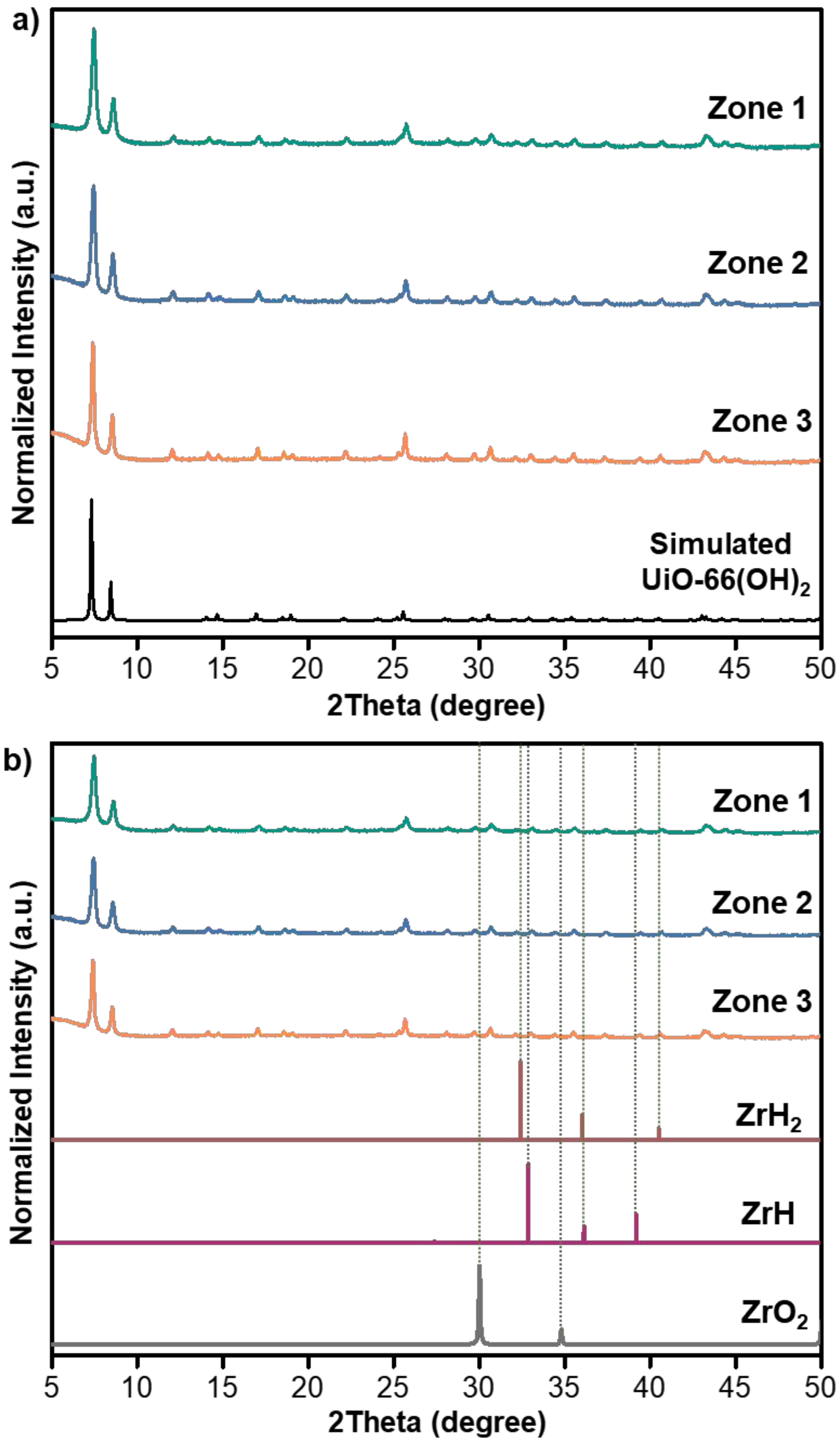

Figure S17. PXRD patterns of UiO-66$(OH)_2$ extracted from the three zones of the tubular reactor following adsorption of 15 ppm of MB dye, a) Comparison with the simulated patterns of UiO-66$(OH)_2$, and b) comparison with the simulated patterns of ZrO_2, ZrH, and ZrH_2.

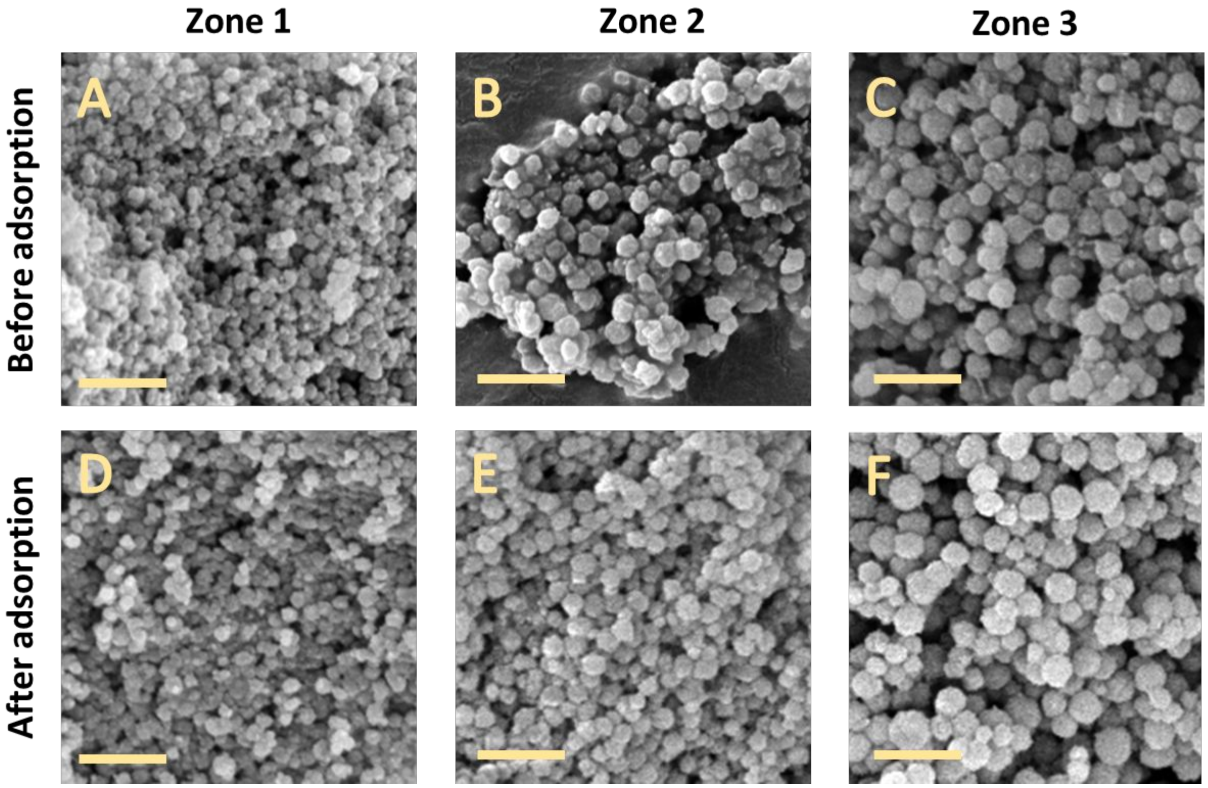

Figure S18. SEM images of UiO-66$(OH)_2$-0 particles isolated from all zones before and after adsorption of 15 ppm of methylene blue. SEM scale bar is 500 nm.

MOF sample	BET surface area before adsorption ($m^2.g^{-1}$)	BET surface area after adsorption ($m^2.g^{-1}$)
UiO-66$(OH)_2$-Z1	201	60
UiO-66$(OH)_2$-Z2	409	67
UiO-66$(OH)_2$-Z3	500	89

Table S5. BET surface area of the three zones of UiO-66$(OH)_2$-0 before and after adsorption of MB.

7. References

1. Jrad, A.; Hmadeh, M.; Awada, G.; Chakleh, R.; Ahmad, M., Efficient biofuel production by MTV-UiO-66 based catalysts. *Chem. Eng. J.* **2021,** *410*, 128237.
2. Wang, Y. L.; Zhang, S.; Zhao, Y. F.; Bedia, J.; Rodriguez, J. J.; Belver, C., UiO-66-based metal organic frameworks for the photodegradation of acetaminophen under simulated solar irradiation. *Journal of Environmental Chemical Engineering* **2021,** *9* (5), 106087.
3. Chen, S.; Liu, J.; Xu, Y.; Li, Z.; Wang, T.; Xu, J.; Wang, Z., Hydrogen storage properties of the novel crosslinked UiO-66-(OH)2. *Int. J. Hydrogen Energy* **2018,** *43* (32), 15370-15377.

4. Eltaweil, A. S.; Abd El-Monaem, E. M.; El-Subruiti, G. M.; Abd El-Latif, M. M.; Omer, A. M., Fabrication of UiO-66/MIL-101(Fe) binary MOF/carboxylated-GO composite for adsorptive removal of methylene blue dye from aqueous solutions. *RSC Adv.* **2020,** *10* (32), 19008-19019.
5. Mohammadi, A. A.; Alinejad, A.; Kamarehie, B.; Javan, S.; Ghaderpoury, A.; Ahmadpour, M.; Ghaderpoori, M., Metal-organic framework Uio-66 for adsorption of methylene blue dye from aqueous solutions. *International Journal of Environmental Science and Technology* **2017,** *14* (9), 1959-1968.
6. Issa, R.; Ibrahim, F. A.; Al-Ghoul, M.; Hmadeh, M., Controlled growth and composition of multivariate metal-organic frameworks-199 via a reaction-diffusion process. *Nano Research* **2021,** *14* (2), 423-431.
7. Li, Y.; Gao, C.; Jiao, J.; Cui, J.; Li, Z.; Song, Q., Selective Adsorption of Metal–Organic Framework toward Methylene Blue: Behavior and Mechanism. *ACS Omega* **2021,** *6* (49), 33961-33968.
8. Wo, R.; Li, Q.-L.; Zhu, C.; Zhang, Y.; Qiao, G.-f.; Lei, K.-y.; Du, P.; Jiang, W., Preparation and Characterization of Functionalized Metal–Organic Frameworks with Core/Shell Magnetic Particles (Fe3O4@SiO2@MOFs) for Removal of Congo Red and Methylene Blue from Water Solution. *Journal of Chemical & Engineering Data* **2019,** *64* (6), 2455-2463.
9. Li, L.; Liu, X. L.; Geng, H. Y.; Hu, B.; Song, G. W.; Xu, Z. S., A MOF/graphite oxide hybrid (MOF: HKUST-1) material for the adsorption of methylene blue from aqueous solution. *Journal of Materials Chemistry A* **2013,** *1* (35), 10292-10299.
10. Zhou, Y.; Qin, L.; Wu, M.-K.; Han, L., A Bifunctional Anionic Metal–Organic Framework: Reversible Photochromism and Selective Adsorption of Methylene Blue. *Crystal Growth & Design* **2018,** *18* (10), 5738-5744.
11. Zhao, X.; Liu, S.; Tang, Z.; Niu, H.; Cai, Y.; Meng, W.; Wu, F.; Giesy, J. P., Synthesis of magnetic metal-organic framework (MOF) for efficient removal of organic dyes from water. *Scientific Reports* **2015,** *5* (1), 11849.
12. Shen, T.; Luo, J.; Zhang, S.; Luo, X., Hierarchically mesostructured MIL-101 metal–organic frameworks with different mineralizing agents for adsorptive removal of methyl orange and methylene blue from aqueous solution. *Journal of Environmental Chemical Engineering* **2015,** *3* (2), 1372-1383.
13. Wang, X.; Zhang, J.; Jiang, J.; Zheng, G.; Li, W., Preparation of bead-like PAN/ZIF-8 nanofiber membrane for methyl blue adsorption by one-step electrospinning. *Mater. Lett.* **2023,** *338*, 134057.
14. Soni, S.; Bajpai, P. K.; Mittal, J.; Arora, C., Utilisation of cobalt doped Iron based MOF for enhanced removal and recovery of methylene blue dye from waste water. *Journal of Molecular Liquids* **2020,** *314*, 113642.
